# Effect of 3-subsitution of quinolinehydroxamic acids on selectivity of histone deacetylase isoforms

**DOI:** 10.1080/14756366.2020.1839446

**Published:** 2020-11-08

**Authors:** Samir Mehndiratta, Mei-Chuan Chen, Yuh-Hsuan Chao, Cheng-Hsin Lee, Jing-Ping Liou, Mei-Jung Lai, Hsueh-Yun Lee

**Affiliations:** a School of Pharmacy, College of Pharmacy, Taipei Medical University, Taipei, Taiwan; b Ph.D. Program in Clinical Drug Development of Herbal Medicine, College of Pharmacy, Taipei Medical University, Taipei, Taiwan; c Traditional Herbal Medicine Research Center of Taipei Medical University Hospital, Taipei, Taiwan; d Biomedical Commercialization Center, Taipei Medical University, Taipei, Taiwan; e Ph.D. Program in Drug Discovery and Development Industry, College of Pharmacy, Taipei Medical University, Taipei, Taiwan

**Keywords:** Quinoline, HDAC, lung cancer, colon cancer, hydroxamic acid, acrylamide

## Abstract

A series of 3-subsituted quinolinehydroxamic acids has been synthesised and evaluated for their effect on human lung cancer cell line (A549), human colorectal cancer cell line (HCT116) and HDAC isoforms 1, 2, 6, and 8. The results indicated that substitution at C3 of quinoline is favoured for HDAC6 selectivity. Two compounds (**25** and **26**) were also found to be potent anti-proliferative compounds with IC_50_ values ranging from 1.29 to 2.13 µM against A549 and HCT116 cells. These compounds displayed remarkable selectivity for HDAC6 over other HDAC isoforms with nanomolar IC_50_ values. Western blot analysis revealed that compounds of this series activate apoptotic caspase pathway as indicated by cleavage of caspase 3, 8, and 9 and also increase phosphorylated H2AX thus inducing DNA double strand fragmentation in a concentration dependent manner. Flow cytometric analysis also displayed a dose dependent increase of cell population in sub G1 phase.

## Graphical Abstract

**Figure:**
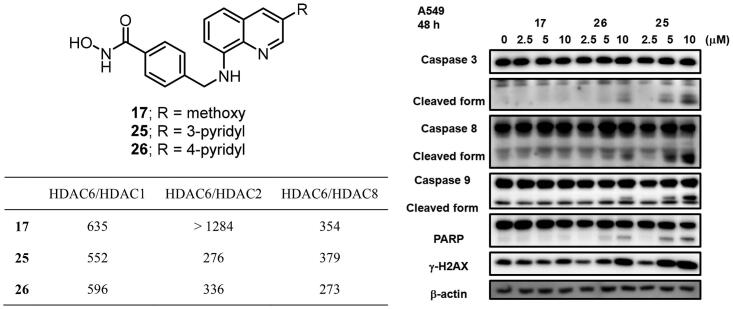

## 1. Introduction

**Figure 1.**
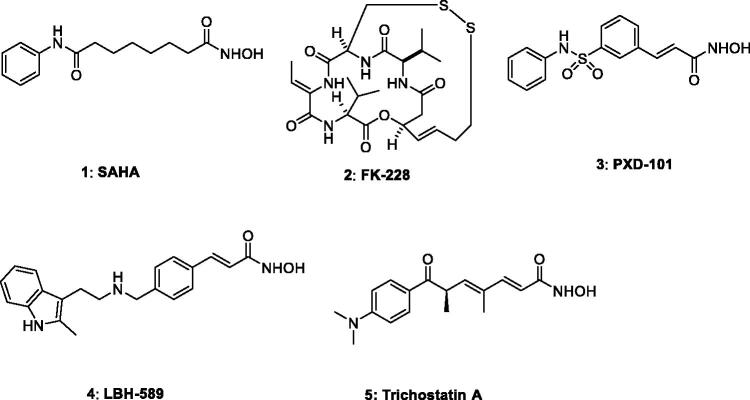
Various examples of histone deacetylase inhibitors (**1**–**5**).

Over last two decades *Epigenetics* have emerged as a potential target for the treatment of cancer and various other physiological disorders. Gene expression regulation by epigenetic modifications play pivotal role and thus have drawn a lot interest for the development of therapeutics capable of altering gene expressions by modulating post-translational changes in histone e.g. acetylation, methylation etc. Histone post-translational modifications act as regulatory marks which are important for the control of transcription and chromatin architecture. Chromatin is a compact structure of nucleosomes and is formed from DNA-wrapped histone proteins. Two enzymes, histone acetylase (HAT) and histone deacetylase (HDAC) together control the acetylation level of lysine residues in the chromatin *N*-terminal region. The acetylation level is correlated with the structure of chromatin. HAT acetylates lysine residues of histone and the resultant neutralised histones loose chromatin, which subsequently results in activation of gene expression. In contrast, HDAC removes acetyl groups and the resulting positively ionised proteins lead to the condensation of chromatin, which represses gene expression. Therefore it can be stated that histone acetylation is carried away by histone transferases (HATs) and histone deacetylases (HDACs) reverse the action of HATs. Epigenetic modification regulates the genetic expression of chromatin without changing its DNA sequence, and the aberrance of this process is highly correlated with the occurrence of disease^1–4^^,^^8^^a^. Consequently, epigenetic regulation has currently become an attractive target for the treatment of a variety of diseases such as cancer^5–8^^b^, inflammation,^9–17^ neurological disorders^18^ and HIV^19–20^. Many HDACs have been reported to be overexpressed in various malignancies and therefore various HDAC inhibitors (HDIs) have been developed to treat these diseases. Since 2006, four HDAC inhibitors have been approved by FDA viz. Vorinostat (SAHA), Romidepsin (FK228), Belinostat (PXD101), and Panobinostat (LBH589). SAHA is the first HDI that received FDA approval in 2006 for the treatment of Cutaneous T cell Lymphoma (CTCL). Many new HDAC inhibitors, including dual/multitarget inhibitors^8^^b^, have been developed and many are now in clinical trials (Figure 1).

Our group has focussed on the development of HDAC inhibitors with various core scaffolds including indole^21–24^, indoline^25^^,^^26^, and quinoline^27^^,^^28^. In a study related of quinoline-derived compounds, we found that 2-(phenylsulfonyl)quinoline-*N*-hydroxyacrylamides (**6**) exhibit potent pan-HDAC inhibitory and antiproliferative activity^27^.

**Figure 2.**
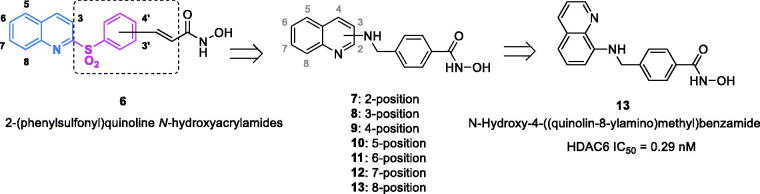
Previously synthesised quinoline-containing HDAC inhibitors (**6–13**).

**Figure 3.**
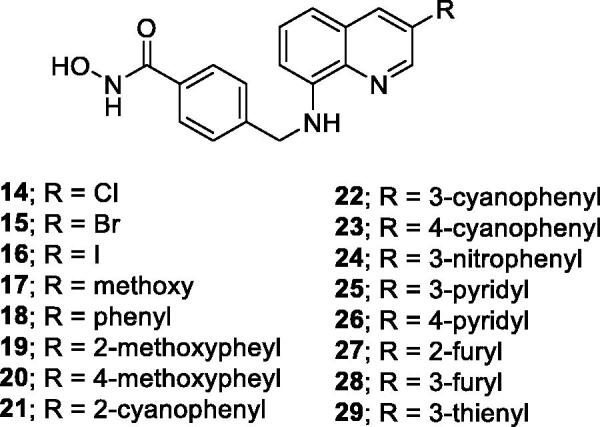
Synthetic 3-substituted quinolinehydroxamic acids (**14–29**).

Recent reports brought our attention to the development of selective inhibitors and on further modifying the lead molecule (**6**), we reported the design and synthesis of *N*-hydroxy-4-((quinolin-8-ylamino)methyl)benzamide (**13**), a selective HDAC6 inhibitor which exhibits potent activity against multiple myeloma (Figure 2)^28^^,^^29^. Continuing our efforts on investigation of quinoline-containing molecules, the current study focuses on the modification of compound **13**, and evaluates the effect of various substituents at C3 of quinoline to generate a series of 3-substituted quinolinehydroxamic acids with selective inhibitory and anti-proliferative activities against HDAC6 (Figure 3).

## 2. Results and discussion

### 2.1. Chemistry

**Scheme 1.**
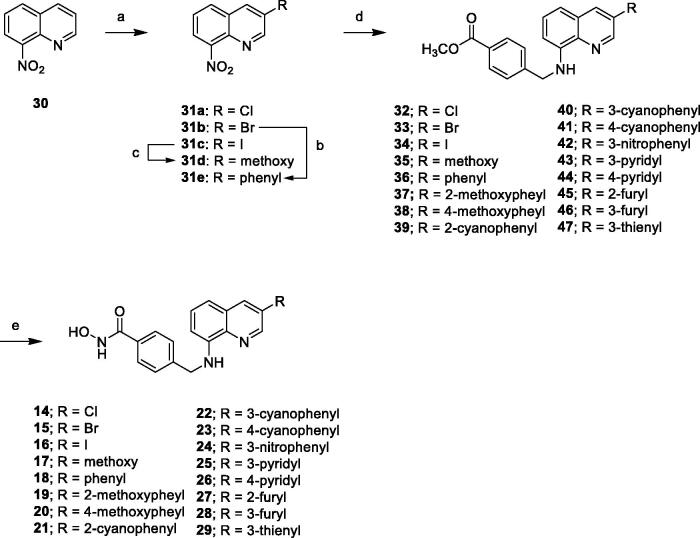
Synthetic Approaches to Compounds **14–29***^a^*. ^a^Reagents and conditions: (a) NXS, acetic acid, 110 ^o^C, 30–61%; (b) phenylboronic acid, Pd(OAc)_2_, PPh_3_, Na_2_CO_3_, THF, H_2_O, MW, 90 ^o^C, 90%; (c) CuI, 1,10-phenanthroline, Cs_2_CO_3_, MeOH, toluene, MW, 70 ^o^C, 57%; (d) for **32–36**: i. Fe powder, 1N HCl_(aq)_, EtOH (or MeOH), reflux; ii. methyl 4-formylbenzoate, NaBH(OAc)_3_, DCM, 40 ^o^C; for **37**–**47**: **33**, boronic acids, Pd(PPh_3_)_4_, TBAB, 2M K_2_CO_3(aq)_, dioxane, reflux, 35–69%; (e) i. 1N LiOH_(aq)_, dioxane, rt; ii. NH_2_OTHP, HBTU, DMF, TEA, rt; iii. 10% TFA_(aq)_, MeOH, rt, 28–55%.

Scheme 1 describes the synthesis of designed compounds (**14–29**), which begins with halogenation of 8-nitroquinoline (**30**) with *N*-halosuccinimide to furnish 3-halogenated 8-nitroquinolines (**31a–31c**). The iodine in compound **31c** was replaced by a methoxy group in the presence of CuI and 1,10-phenanthroline to generate compound **31d**. Meanwhile, compound **31b** underwent a Suzuki arylation with phenylboronic acid to furnish compound **31e**. Subsequently, reduction of the nitro groups of **31a–31e** by iron powder, followed by reductive amination with methyl 4-formylbenzoate in the presence of NaBH(OAc)_3_, led to compounds **32**–**36**. Compound **33** underwent Suzuki arylation with various arylboronic acids to further afford compounds **37–47**. The conversion of **32–47** to the desired hydroxamic acids (**14–29**) was achieved by hydrolysis of the ester by LiOH, amidation with NH_2_OTHP, and TFA-mediated deprotection, reactions that were conducted sequentially.

### 2.2. Biological evaluation

#### 2.2.1. In vitro *cell growth inhibitory activity*

All synthetic compounds (**14–29**) were tested for their anti-proliferative activity in A549 and HCT116 cells, with SAHA (*N*^1^-hydroxy-*N*^8^-phenyloctanediamide) or PXD101 ((*E*)-*N*-hydroxy-3–(3-(*N*-phenylsulfamoyl)phenyl)acrylamide) as reference compounds (Table 1)^27^. Cell line inhibitory results show that halogen-substituted or most aryl/heteroaryl-substituted compounds have similar cellular activity. However, the methoxyphenyl group in **19** and **20** and the 2-cyanophenyl group (**21**) result in diminished activity. Comparison of **21**, **22**, and **23** revealed that the cyano group is disfavoured at C2’ of the C3-phenyl group. Among compounds possessing a C3-heteroaryl group, compounds **25** and **26** with pyridine substituents showed improved antiproliferative activity against A549 and HCT116 cells. Compound **25** in particular, inhibits the growth of A549 and HCT116 cells with IC_50_ values of 1.29 and 1.61 µM, respectively.

**Table 1.** Antiproliferative activity (IC_50_, μM) against human cancer cell lines by compounds **14–29**

	Cell lines	
Compd	A549	HCT116
**14**	3.14 ± 0.31	4.82 ± 0.29
**15**	> 10	> 10
**16**	3.41 ± 0.9	4.79 ± 0.47
**17**	3.11 ± 0.24	4.76 ± 0.43
**18**	4.97 ± 0.06	4.70 ± 0.38
**19**	> 10	7.19 ± 0.77
**20**	9.10 ± 0.30	> 10
**21**	8.65 ± 2.35	6.00 ± 1.56
**22**	2.04 ± 0.35	3.18 ± 0.16
**23**	3.64 ± 0.66	3.50 ± 0.32
**24**	4.05 ± 0.42	2.88 ± 0.21
**25**	1.29 ± 0.41	1.61 ± 0.22
**26**	2.13 ± 0.13	2.03 ± 0.46
**27**	3.18 ± 0.19	2.20 ± 0.17
**28**	3.77 ± 0.28	3.77 ± 0.21
**29**	3.84 ± 0.40	3.59 ± 0.51
SAHA [27]	1.02 ± 0.15	0.15 ± 0.03
PXD101 [27]	0.78 ± 0.07	0.13 ± 0.01

*IC_50_ values higher than 10 μM are estimated based on the best curve fitting available.

#### 2.2.2. HDAC isoform inhibitory activity

The activity of several compounds and reference compounds against trichostatin A ((R,2E,4E)-7–(4-dimethylamino)phenyl)-*N*-hydroxy-4.6-dimethyl-7-oxahepta-2,4-dienamide (**5**) were tested for HDAC isoform selectivity against HDAC1, 2, 6 and 8 (1Table 2). The results from compounds **17**, **25**, and **26** reveals that a polar substituent at C3 shows improvement of HDAC6 selectivity and six-membered aromatic rings at C3 are favoured for HDAC6 selectivity. Compound **25**, with a 3-pyridyl group at C3 of quinoline shows remarkable HDAC6 selectivity over HDAC1, 2, and 8 with selectivity ratios of 552, 276, and 379 respectively (values under IC_50_ in Table 2, presented parenthetically). On the other hand, five-membered heterocycles such as furan (**27**, **28**) and thiophene (**29**), which act as bioisosteres of a phenyl ring, result in decrease of HDAC6 selectivity over that of HDAC 1, 2 and 8.

**Table 2.** Inhibition of the Activity (IC_50_^a^, M) of HDAC Isoforms 1, 2, 6, and 8 (selectivity ratio^b^ is shown in brackets).

	HDAC isoforms			
Compd	HDAC1	HDAC2	HDAC6	HDAC8
**14**	4.32 × 10^−6^	–	2.02 × 10^−8^	3.64 x 10^−6^
	(214)			(180)
**17**	4.95 × 10^−6^	> 10^−5^	7.79 × 10^−9^	2.76 × 10^−6^
	(635)	(> 1284)		(354)
**18**	5.30 × 10^−6^	> 10^−5^	1.94 × 10^−8^	6.88 × 10^−6^
	(273)	(> 515)		(355)
**22**	9.11 × 10^−6^	1.98 × 10^−6^	3.62 × 10^−8^	3.83 × 10^−6^
	(252)	(55)		(106)
**25**	2.62 × 10^−6^	1.31 × 10^−6^	4.75 × 10^−9^	1.80 × 10^−6^
	(552)	(276)		(379)
**26**	5.13 × 10^−6^	2.89 × 10^−6^	8.61 × 10^−9^	2.35 × 10^−6^
	(596)	(336)		(273)
**27**	3.40 × 10^−6^	1.28 × 10^−6^	2.07 × 10^−8^	3.14 × 10^−6^
	(164)	(62)		(152)
**28**	4.59 × 10^−6^	2.10 × 10^−6^	1.34 × 10^−8^	2.91 × 10^−6^
	(343)	(157)		(216)
**29**	6.00 × 10^−6^	3.52 × 10^−6^	3.01 × 10^−8^	4.93 × 10^−6^
	(199)	(117)		(164)
Trichostatin A	1.00 × 10^−8^	2.00 × 10^−8^	2.22 × 10^−9^	6.34 × 10^−7^
	(5)	(9)		(286)

^a^ Dashed line indicates no inhibition or compound activity that could not be fitted to an IC_50_ curve. IC_50_ value higher than 10 μM is estimated based on the best curve fitting available.

^b^ Selectivity ratio: selectivity ratio of HDAC subtypes over HDAC6.

#### 2.2.3. Western blot analysis

**Figure 4.**
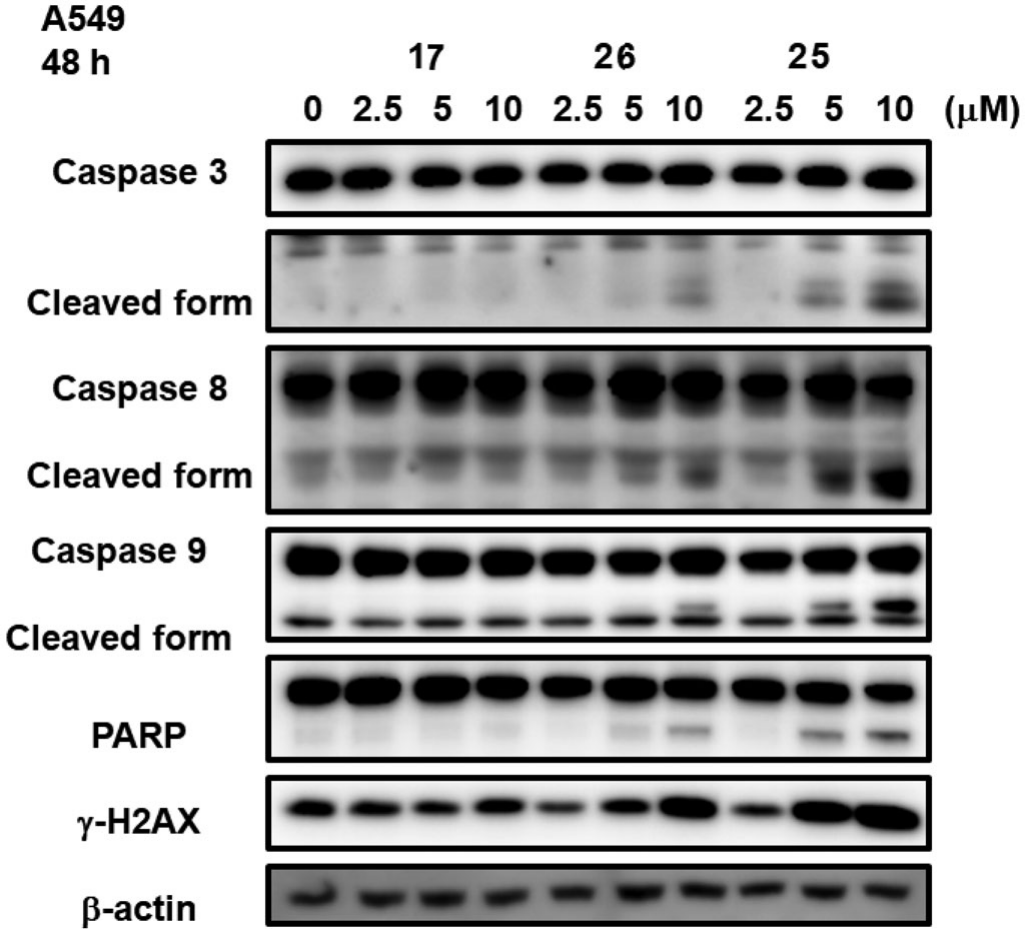
Effect of treatment with compound **17**, **25**, and **26** at three different doses (0.25 µM, 5 µM, and 10 µM) on cleavage of caspase 3, 8, and 9 and PARP. Increased expression of gamma H2AX, a phosporylated form of H2AX, indicates increased DNA double strand fragmentation of A549 cells treated for 48 h in a dose- dependent manner.

Western blot analysis indicated that compounds of this series induce apoptosis by activation of caspase and PARP pathways. Compound **25** with a 3-pyridyl substituent and compound **26** with a 4-pyridyl substitution at C3 of quinoline demonstrated dose dependent increases in caspase 3, 8, and 9 cleavage in A549 cells treated with **17**, **25**, and **26** for 48 h. **23** was found to be the most potent compound amongst these three tested compounds followed by **26** which was further followed by **17**. The order of their efficacy in cleaving caspase isoforms also follows the same order as their *in vitro* cell growth inhibitory activity with **17** being the least potent amongst these three compounds. These molecules also increase dose dependent expression of gamma H2AX indicating increased DNA double strand fragmentation (Figure 4).

#### 2.2.4. Flow cytometry analysis

**Figure 5.**
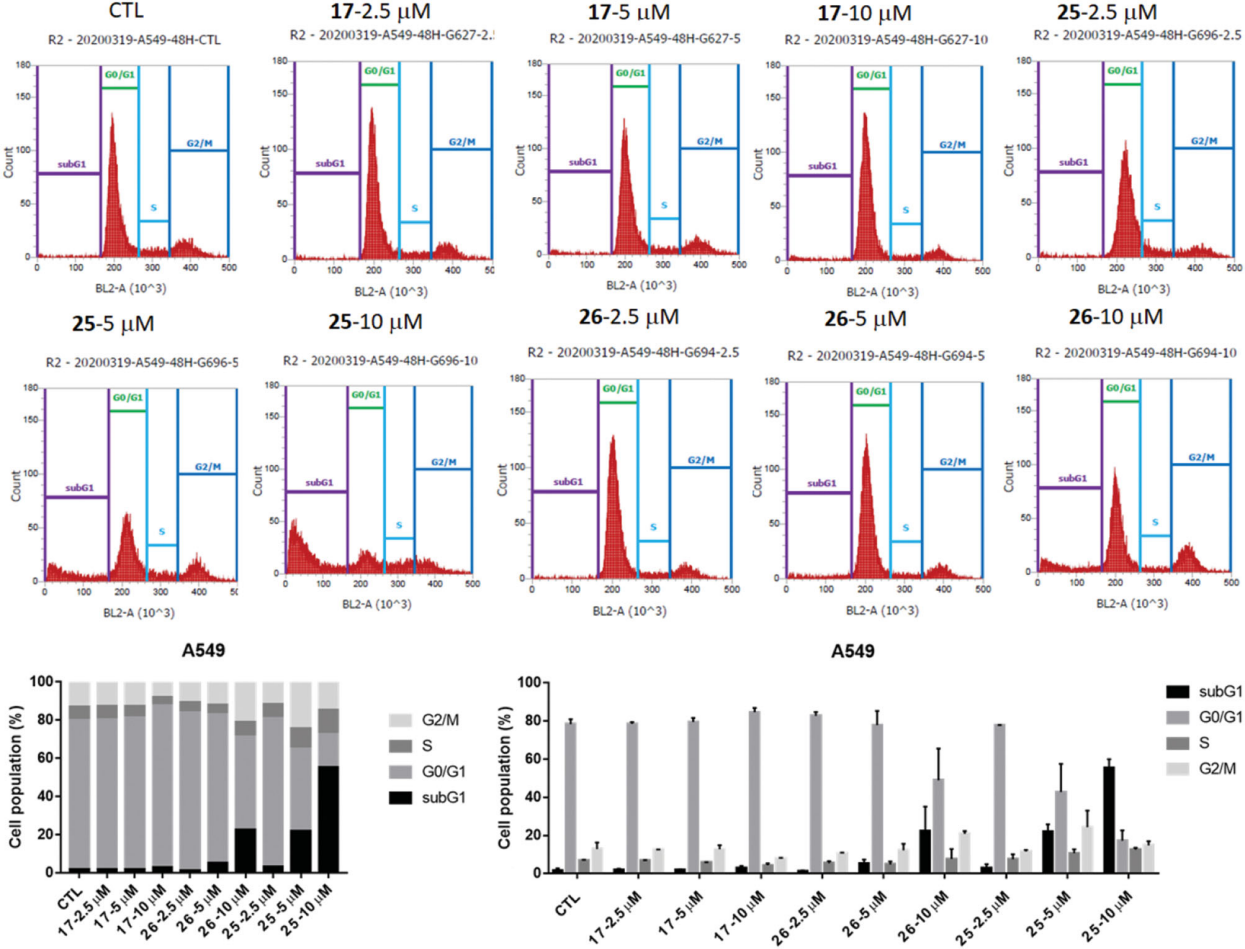
(A) A549 cells were treated with or without **17**, **25**, and **26** (0.25, 0.5, 10 μM) for 48 h and were analysed by flow cytometry for cell cycle distribution. (B) After starvation for 24 h, A549 cells were then treated with compounds for the indicated time. After labelling with propidium iodide, DNA content was analysed by flow cytometry. (C) Quantification of cell population in Sub G1, G0/G1, S and G2/M phase. In A, B, and C, percent of cells = 100%.

The effects of compound **17**, **25**, and **26** on cell cycle progression on A549 cells were examined by flow cytometry. Treatment of A549 cells with **25** and **26** resulted in concentration dependent accumulation of A549 cells in sub G1 phase with concomitant losses from the G0/G1 phase. Compounds followed the same trend in their activity as that of western blot analysis owing to same reason. Furthermore, a characteristic hypo diploid sub G0/1-peak was observed as a consequence of increased apoptosis and partial DNA loss in A549 cells treated with **25** or **26** in a dose dependent manner (Figure 5).

#### 2.2.5. Docking study of compound 17

**Figure 6.**
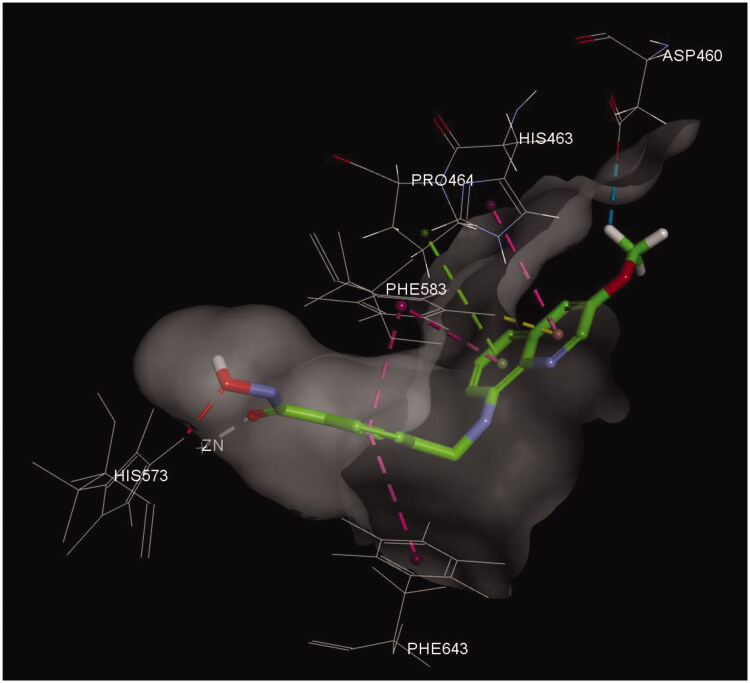
Docking of compound **17** (green) in the binding site of HDAC6 (PDB ID: 6CGP).

Compound **17** possesses the most HDAC6 selectivity over other isoforms; therefore, we docked **17** into the published crystal structure of HDAC6 (PDBID: 6CGP, Figure 6) in an attempt to understand the interaction of compound **17** with HDAC6, which was conducted using *Discovery Studio2017R2*. The hydroxamic acid of **17** interacts with a zinc ion (gray dotted line) and forms a hydrogen bond with His573 (red dotted line) in the bottom of the pocket, which is the typical interaction of hydroxamic acid-containing histone deacetylase inhibitors with HDACs. The central phenyl ring has π–π interactions with Phe583 and Phe643 (violet dotted lines) and the quinoline moiety is able to interact with the surrounding amino acids (His463, Pro464, and Phe583), including π-π (violet dotted lines), π-alkyl (green dotted line), and π-sigma interactions (yellow dotted line). Notably, the C3-methoxy group has an additional carbon hydrogen bond interaction with Asp460 (blue dotted line) in the open area, which probably contributes the higher HDAC6 selectivity.

## 3. Conclusion

This study investigated the effect of C3 substitution of quinolinehydroxamic acids on related biological activities, such as antiproliferative and HDAC inhibitory activity. Synthesised compounds showed potent antitumor activities in A549 cells and HCT116 cells with single digit micromolar activities. Pyridine substitution at C3 of quinoline was found to be optimum for the activity of these compounds. The 3-pyridyl compound (**25**) is more potent than the 4-pyridyl isomer (**26**) and with IC_50_ = 4.75 nM, displayed better HDAC6 selectivity. These compounds exhibit their anticancer effects by induction of apoptosis and by fragmenting DNA double strands as revealed by western blot analysis. The increased population of treated A549 cells in sub G1 phase reveals cell cycle arrest in the sub G1 phase indicating DNA loss and apoptosis. These compounds (**17**, **25**, **26**) are potent and selective HDAC6 inhibitors and are crucial to development of the SAR of quinolinehydroxamic acids and to explore the possibilities of structural modifications to yield compounds with selectivity towards HDAC inhibition.

## 4. Experimental section

### 4.1. Chemistry

Nuclear magnetic resonance spectra were obtained with Bruker DRX-300 spectrometer operating at 300 MHz, with chemical shifts reported in parts per million (ppm, *δ*) downfield from TMS, an internal standard. High-resolution mass spectra (HRMS) were measured with a JEOL (JMS-700) electron impact (EI) mass spectrometer. Purity of the final compounds was determined using a Hitachi 2000 series HPLC system using C-18 column (Agilent ZORBAX Eclipse XDB-C18 5 µm. 4.6 mm × 150 mm) and was found to be ≥ 95% in all cases. Flash column chromatography was carried out using silica gel (Merck Kieselgel 60, No. 9385, 230–400 mesh ASTM). All reactions were done under an atmosphere of dry nitrogen.

*General procedure of halogenation of 8-nitroquinoline* (***31a****–****31c***): A solution of 8-nitroquinoline (1 eq) in AcOH (10 ml) was stirred at 110 °C and *N*-halosuccinimide (1.1–1.4 eq) was added in portions to the solution. The mixture was stirred at 110 °C for 1 h, and then cooled to room temperature (RT). The reaction mixture was poured into H_2_O (50 ml), and the resulting precipitate was collected by filtration and washed with H_2_O. The crude product was purified by flash column chromatography on silica gel eluting with EtOAc/*n*-Hexane to afford desired compound.

*General Procedure of reductive amination (****32****–****36****)*: A mixture of nitro compound (1 eq), iron powder (4 eq), NH_4_Cl (5 eq), and 75% MeOH (10 ml) was heated to reflux. The reaction mixture was filtered and the filtrate was concentrated *in vacuo*, and then extracted with DCM and H_2_O. The combined organic layer was dried over anhydrous MgSO_4_, and concentrated *in vacuo* to afford a crude residue. A mixture of the residue (1 eq), methyl 4-formylbenzoate (1.2 eq), sodium triacetoxyborohydride (1.5 eq), AcOH (2–4 drops), and anhydrous DCM (10 ml) was stirred at RT. The mixture was poured into H_2_O, and then extracted with DCM. The combined organic layer was purified by flash column chromatography on silica gel with EtOAc/*n*-Hexane to afford corresponding ester compound **32**–**36**.

*General procedure for Suzuki arylation (****37****–****47****)*: A mixture of **33** (1 eq), substituted phenylboronic acid (1.2 eq), tetrakis(triphenylphosphine)palladium(0) (0.1 eq), TBAB (0.4 eq), 2 M K_2_CO_3_ (1 ml) and dioxane (10 ml) was stirred and refluxed overnight. Dioxane was removed from the reaction mixture *in vacuo*, and the mixture was extracted with H_2_O and EtOAc. The organic layer was collected and concentrated into an oily residue which was purified via column chromatography on silica gel with EtOAc/*n*-hexane to afford corresponding esters **37**–**47**.

*General procedure for hydroxamic acid synthesis*. A mixture of 1 N LiOH (3 ml) and ester (1 mmol) was stirred at 40 °C for 2 h. The reaction was concentrated under reduced pressure and then H_2_O was added. The mixture was acidified with 3 N HCl to give an off-white precipitate. This off-white solid (1 mmol) was dissolved in DMF (10 ml) and EDC·HCl (1.5 mmol) was added, followed by HOBt hydrate (1.5 mmol) and TEA (3 mmol). After being stirred at RT for 30 min, NH_2_OTHP (1.2 mmol) was added and allowed to stir for an additional 5 h. The reaction mixture was quenched with H_2_O and was extracted with EtOAc (25 ml × 3). The combined organic layer was collected, dried over anhydrous MgSO_4_ and concentrated under reduced pressure to give a light yellow residue, which was purified by silica gel chromatography (EtOAc: *n*-hexane = 1:1) to give a colourless liquid. 10% TFA (5 ml) was added to the resulting product dissolved in MeOH (5 ml) and the mixture was stirred at RT for 5 h. The reaction mixture was concentrated under reduced pressure to give a white residue, which was recrystallized from MeOH to afford the desired compound.

#### 4.1.1. 3-Chloro-8-nitroquinoline (31a)

Using *N*-chlorosuccinimide (1.4 eq) and following general procedure of halogenation, the title compound (**31a**) was obtained in 30% yield from **30**: ^1^H NMR (300 MHz, CDCl_3_) δ 7.67 (dd, *J* = 7.8, 8.4 Hz, 1H), 7.98 (dd, *J* = 1.5, 8.4 Hz, 1H), 8.04 (dd, *J* = 1.2, 7.5 Hz, 1H), 8.25 (d, *J* = 2.1 Hz, 1H), 8.97 (d, *J* = 2.4 Hz, 1H); ^13 ^C NMR (75 MHz, CDCl_3_) δ 124.0, 126.8, 129.3, 130.6, 131.3, 134.1, 137.5, 148.3, 151.9.

#### 4.1.2. 3-Bromo-8-nitro-quinoline (31 b)

Using *N*-bromosuccinimide (1.2 eq) and following general procedure of halogenation, the title compound (**31 b**) was obtained in 40% yield from **30**: ^1^H NMR (300 MHz, CDCl_3_) δ 7.67 (dd, *J* = 7.5, 8.1 Hz, 1H), 7.97 (dd, *J* = 1.5, 8.4 Hz, 1H), 8.06 (dd, *J* = 1.5, 7.5 Hz, 1H), 8.44 (d, *J* = 2.1 Hz, 1H), 9.06 (d, *J* = 2.1 Hz, 1H); ^13 ^C NMR (75 MHz, CDCl_3_) δ 119.3, 124.1, 126.7, 129.8, 131.2, 137.4, 137.6, 148.3, 153.7.

#### 4.1.3. 3-Iodo-8-nitro-quinoline (31c)

Using *N*-Iodosuccinimide (1.1 eq) and following general procedure of halogenation, the title compound (**31c**) was obtained in 61% yield from **30**: ^1^H NMR (300 MHz, CDCl_3_) δ 7.62–7.67 (m, 1H), 7.93 (dd, *J* = 1.5, 8.1 Hz, 1H), 8.05 (dd, *J* = 1.2, 7.5 Hz, 1H), 8.65 (d, *J* = 2.1 Hz, 1H), 9.17 (d, *J* = 2.1 Hz, 1H); ^13 ^C NMR (75 MHz, CDCl_3_) δ 92.1, 124.3, 126.5, 130.3, 131.1, 137.6, 143.8, 148.2, 157.9.

#### 4.1.4. 3-Methoxy-8-nitro-quinoline (31d)

A mixture of **31c** (0.10 g, 0.33 mmol), CuI (0.006 g, 0.03 mmol), 1,10-phenanthroline (0.011 g, 0.06 mmol), Cs_2_CO_3_ (0.16 g, 0.50 mmol), MeOH (1 ml), and toluene (0.5 ml) was placed in a 10 ml reaction vessel with a magnetic stirring bar. The vessel was sealed and placed in the microwave cavity. The reaction condition was held at 70 °C for 30 min. After it was cooled to RT, the reaction mixture was filtered and the filtrate was concentrated *in vacuo*, and then extracted with DCM and H_2_O. The combined organic layer was purified by flash column chromatography on silica gel with EtOAc/*n*-hexane to afford compound **31d** (0.04 g, 57%): ^1^H NMR (300 MHz, CDCl_3_) δ 3.98 (s, 3H), 7.43 (d, *J* = 2.7 Hz, 1H), 7.55 (dd, *J* = 7.8, 8.1 Hz, 1H), 7.85 (dd, *J* = 1.5, 7.5 Hz, 1H), 7.92 (dd, *J* = 1.5, 8.4 Hz, 1H), 8.79 (d, *J* = 3.0 Hz, 1H); ^13 ^C NMR (75 MHz, CDCl_3_) δ 55.8, 112.1, 121.0, 126.0, 130.2, 130.9, 134.5, 147.0, 148.4, 154.3.

#### 4.1.5. 8-Nitro-3-phenyl-quinoline (31e)

A mixture of **31 b** (0.30 g, 1.19 mmol), phenylboronic acid (0.29 g, 2.38 mmol), palladium (II) acetate (0.027 g, 0.12 mmol), triphenylphosphine (0.12 g, 0.48 mmol), sodium carbonate (0.25 g, 1.78 mmol), THF (10 ml), and H_2_O (1 ml) was placed into 35 ml reaction vessel with a magnetic stirring bar. The vessel was sealed and placed in the microwave cavity. The temperature was held at 90 °C for 20 min. After it cooled to RT, the reaction mixture was filtered and the filtrate was concentrated *in vacuo*; then extracted with DCM and H_2_O. The combined organic layer was purified by flash column chromatography on silica gel with EtOAc/*n*-hexane to afford compound **31e** (0.27 g, 90%): ^1^H NMR (300 MHz, CDCl_3_) δ 7.45–7.57 (m, 3H), 7.60–7.66 (m, 1H), 7.68–7.71 (m, 2H), 8.03 (d, *J* = 7.2 Hz, 1H), 8.09 (d, *J* = 8.4 Hz, 1H), 8.37 (s,1H), 9.32 (s, 1H); ^13 ^C NMR (75 MHz, CDCl_3_) δ 123.7, 125.8, 127.5, 128.9, 129.0, 129.5, 132.4, 133.1, 135.7, 136.7, 138.5, 148.2, 152.3.

#### 4.1.6. 4-[(3-Chloro-quinolin-8-ylamino)-methyl]-benzoic acid methyl ester (32)

The title compound was obtained in 51% yield from compound **31a** following the general procedure for reductive amination: ^1^H NMR (300 MHz, CDCl_3_) δ 3.91 (s, 3H), 4.62 (d, *J* = 6.0 Hz, 2H), 6.54 (d, *J* = 7.5 Hz, 1H), 6.63 (t, *J* = 5.4 Hz, 1H), 6.98 (d, *J* = 8.4 Hz, 1H), 7.32 (t, *J* = 7.8 Hz, 1H), 7.48 (d, *J* = 8.1 Hz, 2H), 8.00 (d, *J* = 8.4 Hz, 2H), 8.04 (d, *J* = 2.4 Hz, 1H), 8.62 (d, *J* = 2.4 Hz, 1H); ^13 ^C NMR (75 MHz, CDCl_3_) δ 47.4, 52.2, 105.7, 113.7, 127.1, 129.2, 129.3, 130.1, 134.2, 136.1, 144.4, 144.5, 146.1, 167.0.

#### 4.1.7. 4-[(3-Bromo-quinolin-8-ylamino)-methyl]-benzoic acid methyl ester (33)

The title compound was obtained in 68% yield from compound **31 b** following the general procedure for reductive amination: ^1^H NMR (300 MHz, CDCl_3_) δ 3.91 (s, 3H), 4.62 (s, 2H), 6.56 (dd, *J* = 0.6, 7.5 Hz, 1H), 6.98 (dd, *J* = 0.9, 8.4 Hz, 1H), 7.32 (t, *J* = 7.8 Hz, 1H), 7.48 (d, *J* = 8.4 Hz, 2H), 8.01 (d, *J* = 8.4 Hz, 2H), 8.23 (d, *J* = 2.1 Hz, 1H), 8.71 (d, *J* = 2.1 Hz, 1H); ^13 ^C NMR (75 MHz, CDCl_3_) δ 47.4, 52.2, 105.8, 113.6, 117.9, 127.1, 129.1, 129.2, 129.8, 130.1, 1136.1, 137.4, 144.4, 144.5, 147.8. 167.0.

#### 4.1.8. 4-[(3-Iodo-quinolin-8-ylamino)-methyl]-benzoic acid methyl ester (34)

The title compound was obtained in 64% yield from compound **31c** following the general procedure for reductive amination: ^1^H NMR (300 MHz, CDCl_3_) δ 3.90 (s, 3H), 4.59 (s, 2H), 6.55 (dd, *J* = 1.2, 7.8 Hz, 1H), 6.92 (dd, *J* = 1.2, 8.4 Hz, 1H), 7.29 (t, *J* = 8.1 Hz, 1H), 7.45 (d, *J* = 8.4 Hz, 2H), 8.00 (dt, *J* = 1.8, 6.6 Hz, 2H), 8.41 (d, *J* = 2.1 Hz, 1H), 8.82 (d, *J* = 2.1 Hz, 1H); ^13 ^C NMR (75 MHz, CDCl_3_) δ 47.4, 52.2, 90.6, 106.0, 113.5, 127.1, 128.9, 129.2, 130.1, 130.5, 136.2, 143.8, 144.4, 144.5, 152.1, 167.0.

#### 4.1.9. 4-[(3-Methoxy-quinolin-8-ylamino)-methyl]-benzoic acid methyl ester (35)

The title compound was obtained in 63% yield from compound **31d** following the general procedure for reductive amination: ^1^H NMR (300 MHz, CDCl_3_) δ 3.90 (s, 3H), 3.93 (s, 3H), 4.61 (s, 2H), 6.43 (dd, *J* = 1.2, 7.8 Hz, 1H), 6.62 (brs, 1H), 6.98 (dd, *J* = 0.9, 8.4 Hz, 1H), 7.27 (t, *J* = 7.8 Hz, 2H), 7.33 (d, *J* = 2.7 Hz, 1H), 7.49 (dt, *J* = 1.8, 8.1 Hz, 2H), 8.49 (d, *J* = 2.7 Hz, 1H); ^13 ^C NMR (75 MHz, CDCl_3_) δ 47.5, 52.1, 55.6, 103.7, 113.1, 113.9, 127.2, 128.6, 129.1, 129.5, 130.0, 133.2, 140.6, 144.4, 145.0, 153.9, 167.1.

#### 4.1.10. 4-[(3-Phenyl-quinolin-8-ylamino)-methyl]-benzoic acid methyl ester (36)

The title compound was obtained in 45% yield from compound **31e** following the general procedure for reductive amination: ^1^H NMR (300 MHz, CDCl_3_) δ 3.91 (s, 3H), 4.65 (s, 2H), 6.57 (d, *J* = 7.5 Hz, 1H), 6.73 (brs, 1H), 7.14 (dd, *J* = 1.2, 8.1 Hz), 7.34 (t, *J* = 8.1 Hz, 1H), 7.40–7.47 (m, 1H), 7.50–7.55 (m, 4H), 7.70–7.73 (m, 2H), 8.01–8.06 (m, 2H), 8.23 (d, *J* = 2.1 Hz, 1H), 9.01 (d, *J* = 2.4 Hz, 1H); ^13 ^C NMR (75 MHz, CDCl_3_) δ 47.5, 52.1, 105.5, 114.9, 127.2, 127.5, 128.1,128.3, 128.5, 129.1, 129.2, 130.1, 133.6, 134.3, 137.2, 138.2, 144.3, 144.9, 146.4, 167.1.

#### 4.1.11. Methyl 4-(((3–(2-methoxyphenyl)quinolin-8-yl)amino)methyl)benzoate (37)

The title compound was obtained in 35% yield from compound **33** followed the general procedure for Suzuki arylation: ^1^H NMR (300 MHz, CDCl_3_) δ 3.85 (s, 3H), 3.92 (s, 3H), 4.65 (s, 2H), 6.58 (dd, *J* = 0.9, 7.8 Hz, 1H), 7.05 (d, *J* = 8.1 Hz, 1H), 7.13 (d, *J* = 8.4 Hz, 2H), 7.33 (d, *J* = 7.8 Hz, 1H), 7.41–7.46 (m, 2H), 7.52 (d, *J* = 8.4 Hz, 2H), 8.04 (d, *J* = 8.4 Hz, 2H), 8.20 (d, *J* = 2.1 Hz, 1H), 8.98 (d, *J* = 2.1 Hz, 1H).

#### 4.1.12. Methyl 4-(((3–(4-methoxyphenyl)quinolin-8-yl)amino)methyl)benzoate (38)

The title compound was obtained in 38% yield from compound **33** followed the general procedure for Suzuki arylation: ^1^H NMR (300 MHz, CDCl_3_) δ 3.90 (s, 6H), 4.71 (s, 2H), 6.66 (d, *J* = 7.8 Hz, 1H), 7.08 (d, *J* = 8.7 Hz, 2H), 7.20 (d, *J* = 8.1 Hz, 1H), 7.43 (t, *J* = 7.8 Hz, 1H), 7.53 (d, *J* = 8.1 Hz, 2H), 7.66 (d, *J* = 8.7 Hz, 2H), 8.01 (d, *J* = 8.4 Hz, 2H), 8.49 (s, 1H), 9.03 (d, *J* = 2.1 Hz, 1H).

#### 4.1.13. Methyl 4-(((3–(2-cyanophenyl)quinolin-8-yl)amino)methyl)benzoate (39)

The title compound was obtained in 45% yield from compound **33** following the general procedure for Suzuki arylation: ^1^H NMR (300 MHz, CDCl_3_) δ 3.91 (s, 3H), 4.66 (s, 2H), 6.62 (dd, *J* = 0.9, 7.5 Hz, 1H), 7.16 (dd, *J* = 0.9, 8.4 Hz, 1H), 7.38 (t, *J* = 7.8 Hz, 1H), 7.50–7.56 (m, 3H), 7.62 (d, *J* = 7.2 Hz, 1H), 7.73 (td, *J* = 1.5, 7.7 Hz, 1H), 7.85 (td, *J* = 1.2, 7.8 Hz, 1H), 8.02 (d, *J* = 8.4 Hz, 2H), 8.31 (d, *J* = 2.1 Hz, 1H), 8.90 (d, *J* = 2.4 Hz, 1H).

#### 4.1.14. Methyl 4-(((3–(3-cyanophenyl)quinolin-8-yl)amino)methyl)benzoate (40)

The title compound was obtained in 59% yield from compound **33** following the general procedure for Suzuki arylation: ^1^H NMR (300 MHz, CDCl_3_) δ 3.91 (s, 3H), 4.66 (s, 2H), 6.62 (d, *J* = 7.2 Hz, 1H), 7.16 (d, *J* = 8.1 Hz, 1H), 7.38 (t, *J* = 7.8 Hz, 1H), 7.51 (d, *J* = 8.4 Hz, 2H), 7.64 (t, *J* = 7.5 Hz, 1H), 7.73 (d, *J* = 7.8 Hz, 1H), 7.93 (d, *J* = 7.8 Hz, 1H), 7.98–8.03 (m, 3H), 8.26 (d, *J* = 2.1 Hz, 1H), 8.94 (d, *J* = 2.1 Hz, 1H).

#### 4.1.15. Methyl 4-(((3–(4-cyanophenyl)quinolin-8-yl)amino)methyl)benzoate (41)

The title compound was obtained in 51% yield from compound **33** following the general procedure for Suzuki arylation: ^1^H NMR (300 MHz, CDCl_3_) δ 3.91 (s, 3H), 4.69 (s, 2H), 6.67 (d, *J* = 7.8 Hz, 1H), 7.19 (d, *J* = 8.4 Hz, 1H), 7.43 (t, *J* = 8.1 Hz, 1H), 7.52 (d, *J* = 8.4 Hz, 2H), 7.83 (m, 1H), 8.02 (d, *J* = 8.1 Hz, 2H), 8.39 (s, 1H), 9.00 (d, *J* = 2.4 Hz, 1H).

#### 4.1.16. Methyl 4-(((3–(3-nitrophenyl)quinolin-8-yl)amino)methyl)benzoate (42)

The title compound was obtained in 61% yield from compound **33** followed the general procedure for Suzuki arylation: ^1^H NMR (300 MHz, CDCl_3_) δ 3.90 (s, 3H), 4.70 (s, 2H), 6.70 (d, *J* = 7.8 Hz, 1H), 7.22 (s, 1H), 7.46 (t, *J* = 8.1 Hz, 1H), 7.52 (d, *J* = 8.4 Hz, 2H), 7.75 (t, *J* = 8.1 Hz, 1H), 8.00–8.06 (m, 3H), 8.34 (d, *J* = 8.1 Hz, 1H), 8.51 (s, 1H), 8.59 (s, 1H), 9.05 (d, *J* = 2.4 Hz, 1H).

#### 4.1.17. Methyl 4-(((3-(pyridin-3-yl)quinolin-8-yl)amino)methyl)benzoate (43)

The title compound was obtained in 69% yield from compound **33** following the general procedure for Suzuki arylation: ^1^H NMR (300 MHz, CDCl_3_) δ3.91 (s, 3H), 4.67 (s, 2H), 6.64 (d, *J* = 7.5 Hz, 1H), 7.17 (d, *J* = 7.8 Hz, 1H), 7.40 (t, *J* = 8.1 Hz, 1H), 7.51 (d, *J* = 8.4 Hz, 2H), 7.61 (m, 1H), 8.02 (d, *J* = 8.1 Hz, 2H), 8.19 (d, *J* = 6.6 Hz, 1H), 8.31 (s, 1H), 8.73 (s, 1H), 8.98 (s, 1H), 9.02 (s, 1H).

#### 4.1.18. Methyl 4-(((3-(pyridin-3-yl)quinolin-8-yl)amino)methyl)benzoate (44)

The title compound was obtained in 62% yield from compound **33** following the general procedure for Suzuki arylation: ^1^HNMR (300 MHz, CDCl_3_) δ3.91 (s, 3H), 4.68 (s, 2H), 6.69 (d, *J* = 7.8 Hz, 1H), 7.20 (d, *J* = 7.8 Hz, 1H), 7.44 (t, *J* = 7.8 Hz, 1H), 7.51 (d, *J* = 8.1 Hz, 2H), 8.03 (m, 3H), 8.44 (s, 1H), 8.83 (s, 2H), 9.05 (s, 1H).

#### 4.1.19. Methyl 4-(((3-(furan-2-yl)quinolin-8-yl)amino)methyl)benzoate (45)

The title compound was obtained in 40% yield from compound **33** following the general procedure for Suzuki arylation: ^1^H NMR (300 MHz, CDCl_3_) δ 3.90 (s, 3H), 4.63 (s, 2H), 6.53 (d, *J* = 0.6 Hz, 1H), 6.55 (dd, *J* = 1.8, 3.3 Hz, 1H), 6.85 (d, *J* = 3.3 Hz, 1H), 7.10 (d, *J* = 8.1 Hz, 1H), 7.31 (t, *J* = 7.8 Hz, 1H), 7.50 (d, *J* = 8.1 Hz, 2H), 7.57 (d, *J* = 1.5 Hz, 1H), 8.01 (d, *J* = 8.1 Hz, 2H), 8.30 (d, *J* = 2.1 Hz, 1H), 9.04 (d, *J* = 2.1 Hz, 1H).

#### 4.1.20. Methyl 4-(((3-(furan-3-yl)quinolin-8-yl)amino)methyl)benzoate (46)

The title compound was obtained in 37% yield from compound **33** following the general procedure for Suzuki arylation: ^1^H NMR (300 MHz, CDCl_3_) δ 3.91 (s, 3H), 4.65 (s, 2H), 6.56 (d, *J* = 7.8 Hz, 1H), 6.83 (dd, *J* = 0.9, 1.8 Hz, 1H), 7.09 (d, *J* = 8.1 Hz, 1H), 7.33 (t, *J* = 8.1 Hz, 1H), 7.51 (d, *J* = 8.4 Hz, 2H), 7.57 (t, *J* = 1.8 Hz, 1H), 7.91 (s, 1H), 8.01 (d, *J* = 8.4 Hz, 2H), 8.15 (d, *J* = 1.8 Hz, 1H), 8.91 (d, *J* = 2.1 Hz, 1H).

#### 4.1.21. Methyl 4-(((3-(thiophen-3-yl)quinolin-8-yl)amino)methyl)benzoate (47)

The title compound was obtained in 36% yield from compound **33** following the general procedure for Suzuki arylation: ^1^H NMR (300 MHz, CDCl_3_) δ 3.91 (s, 3H), 4.63 (s, 2H), 6.54 (d, *J* = 7.5 Hz, 1H), 6.72 (s, 1H), 7.10 (d, *J* = 8.1 Hz, 1H), 7.32 (t, *J* = 7.8 Hz, 1H), 7.46–7.51 (m, 4H), 7.62 (s, 1H), 8.02 (d, *J* = 8.4 Hz, 2H), 8.20 (d, *J* = 1.8 Hz, 1H), 9.01 (d, *J* = 2.1 Hz, 1H).

#### 4.1.2 Synthesis of hydroxamic acids 14–29

##### 4.1.2.1. 4-[(3-Chloro-quinolin-8-ylamino)-methyl]-N-hydroxy-benzamide (14)

The title compound was obtained in 33% overall yield from compound **32** following the general procedure for hydroxamic acid synthesis: mp 188.3–188.9 °C; ^1^H NMR (300 MHz, DMSO-d_6_) δ 4.58 (d, *J* = 6.3 Hz, 2H), 6.54 (d, *J* = 7.5 Hz, 1H), 7.03 (d, *J* = 7.8 Hz, 1H), 7.29–7.34 (m, 2H), 7.45 (d, *J* = 8.1 Hz, 2H), 7.70 (d, *J* = 8.1 Hz, 2H), 8.38 (d, *J* = 2.4 Hz, 1H), 8.73 (d, *J* = 2.4 Hz, 1H), 8.99 (s, 1H), 11.14 (s, 1H); ^13 ^C NMR (75 MHz, DMSO-d_6_) δ 45.7, 105.4, 112.7, 126.9, 127.0, 128.0, 128.9, 129.2, 131.4, 134.1, 135.6, 143.1, 144.3, 145.4, 164.2; HRMS (ESI) for C_17_H_15_ClN_3_O_2_ (M + H^+^) calcd 328.0853, found 328.0849; HPLC purity of 100.00% (retention time = 29.63).

##### 4.1.2.2. 4-[(3-Bromo-quinolin-8-ylamino)-methyl]-N-hydroxy-benzamide (15)

The title compound was obtained in 30% overall yield from compound **33** following the general procedure for hydroxamic acid synthesis: mp 188.5–189.2 °C; ^1^H NMR (300 MHz, DMSO-d_6_) δ 4.58 (d, *J* = 6.3 Hz, 2H), 6.55 (d, *J* = 7.8 Hz, 1H), 7.02 (dd, *J* = 0.6, 8.1 Hz, 1H), 7.29–7.34 (m, 2H), 7.44 (d, *J* = 8.1 Hz, 2H), 7.68 (d, *J* = 8.4 Hz, 2H), 8.55 (d, *J* = 2.4 Hz, 1H), 8.79 (d, *J* = 2.4 Hz, 1H), 8.97 (s, 1H), 11.12 (s, 1H); ^13 ^C NMR (75 MHz, DMSO-d_6_) δ 45.7, 105.5, 112.6, 117.2, 126.9, 127.0, 129.2, 129.6, 131.4, 135.6, 137.3, 143.1, 144.3, 147.2, 164.2; HRMS (ESI) for C_17_H_15_BrN_3_O_2_ (M + H^+^) calcd 372.0348, found 372.0343; HPLC purity of 98.75% (retention time = 30.55).

##### 4.1.2.3. N-Hydroxy-4-[(3-iodo-quinolin-8-ylamino)-methyl]-benzamide (16)

The title compound was obtained in 29% overall yield from compound **34** following the general procedure for hydroxamic acid synthesis: mp 193.8–194.7 °C; ^1^H NMR (300 MHz, DMSO-d_6_) δ 4.57 (d, *J* = 6.3 Hz, 2H), 6.53 (dd, *J* = 1.2, 7.8 Hz, 1H), 6.98 (dd, *J* = 0.9, 8.1 Hz, 1H), 7.25–7.31 (m, 2H), 7.44 (d, *J* = 8.1 Hz, 2H), 7.67 (d, *J* = 8.1 Hz, 2H), 8.69 (d, *J* = 2.1 Hz, 1H), 8.88 (d, *J* = 2.1 Hz, 1H), 8.97 (s, 1H), 11.12 (s, 1H); ^13 ^C NMR (75 MHz, DMSO-d_6_) δ 45.7, 91.4, 105.5, 112.5, 126.9, 127.0, 128.8, 130.2, 131.4, 135.7, 143.2, 143.4, 144.3, 151.7, 164.2; HRMS (ESI) for C_17_H_15_IN_3_O_2_ (M + H^+^) calcd 420.0209, found 420.0203; HPLC purity of 95.67% (retention time = 31.33).

##### 4.1.2.4. N-Hydroxy-4-[(3-methoxy-quinolin-8-ylamino)-methyl]-benzamide (17)

The title compound was obtained in 30% overall yield from compound **35** following the general procedure for hydroxamic acid synthesis: mp 171.8–172.9 °C; ^1^H NMR (300 MHz, DMSO-d_6_) δ 3.91 (s, 3H), 4.55 (d, *J* = 6.3 Hz, 2H), 6.37 (d, *J* = 7.2 Hz, 1H), 7.14 (t, *J* = 6.3 Hz, 1H), 7.21 (t, *J* = 8.1 Hz, 1H), 7.45 (d, *J* = 8.1 Hz, 2H), 7.61 (d, *J* = 2.7 Hz, 1H), 7.68 (d, *J* = 8.1 Hz, 2H), 8.49 (d, *J* = 2.7 Hz, 1H), 8.98 (s, 1H), 11.13 (s, 1H); ^13 ^C NMR (75 MHz, DMSO-d_6_) δ 45.9, 55.5, 103.0, 112.8, 113.2, 126.9, 127.0, 128.4, 129.2, 131.3, 132.5, 139.9, 143.5, 144.2, 153.4, 164.2; HRMS (ESI) for C_18_H_18_N_3_O_3_ (M + H^+^) calcd 324.1348, found 324.1349; HPLC purity of 97.57% (retention time = 25.47).

##### 4.1.2.5. N-Hydroxy-4-[(3-phenyl-quinolin-8-ylamino)-methyl]-benzamide (18)

The title compound was obtained in 40% overall yield from compound **36** following the general procedure for hydroxamic acid synthesis: mp 172.7–173.2 °C; ^1^H NMR (300 MHz, DMSO-d_6_) δ 4.61 (d, *J* = 6.3 Hz, 2H), 6.53 (d, *J* = 7.5 Hz, 1H), 7.14 (d, *J* = 7.8 Hz, 1H), 7.27–7.33 (m, 2H), 7.40–7.60 (m, 5H), 7.70 (d, *J* = 8.1 Hz, 2H), 7.87 (d, *J* = 7.2 Hz, 2H), 8.48 (d, *J* = 2.1 Hz, 1H), 8.99 (s, 1H), 9.09 (d, *J* = 2.1 Hz, 1H), 11.15 (s, 1H); ^13 ^C NMR (75 MHz, DMSO-d_6_) δ 45.8, 105.0, 113.9, 126.9, 127.0, 127.2, 128.1, 129.2, 131.4, 133.0, 133.2, 136.7, 137.2, 143.4, 144.1, 145.8, 164.2; HRMS (ESI) for C_23_H_20_N_3_O_2_ (M + H^+^) calcd 370.1556, found 370.1557; HPLC purity of 97.59% (retention time = 33.67).

##### 4.1.2.6. N-Hydroxy-4-(((3–(2-methoxyphenyl)quinolin-8-yl)amino)methyl)benzamide (19)

The title compound was obtained in 45% overall yield from compound **37** following the general procedure for hydroxamic acid synthesis: ^1^H NMR (300 MHz, DMSO-d_6_) δ 3.84 (s, 3H), 4.64 (s, 2H), 6.57 (d, *J* = 0.9 Hz, 1H), 6.60–7.11 (m, 2H), 7.14 (d, *J* = 7.8 Hz, 1H), 7.27 (t, *J* = 8.1 Hz, 1H), 7.42 (d, *J* = 7.5 Hz, 2H), 7.52 (d, *J* = 8.1 Hz, 2H), 7.71 (d, *J* = 8.4 Hz, 2H), 8.18 (d, *J* = 2.4 Hz, 1H), 8.86 (d, *J* = 2.1 Hz, 1H); ^13^C NMR (75 MHz, DMSO-d_6_) δ 45.9, 55.6, 105.0, 111.9, 113.7, 121.1, 126.6, 126.9, 127.0, 128.0, 129.8, 130.7, 131.4, 131.7, 135.3, 136.1, 143.4, 144.0, 148.0, 156.4, 164.2; HRMS (EI) for C_24_H_22_N_3_O_3_(M + H^+^) calcd 400.1661, found 400.1662; HPLC purity of 93.21% (retention time = 32.91).

##### 4.1.2.7. N-Hydroxy-4-(((3–(4-methoxyphenyl)quinolin-8-yl)amino)methyl)benzamide (20)

The title compound was obtained in 37% overall yield from compound **38** following the general procedure for hydroxamic acid synthesis: ^1^H NMR (300 MHz, DMSO-d_6_) δ 3.83 (s, 3H), 4.61 (s, 2H), 6.51 (dd, *J* = 0.9 Hz, 7.5 Hz, 1H), 7.10–7.13 (m, 3H), 7.28 (t, *J* = 8.1 Hz, 1H), 7.47 (d, *J* = 8.1 Hz, 2H), 7.69 (d, *J* = 8.1 Hz, 2H), 7.81–7.85 (m, 2H), 8.41 (d, *J* = 2.1 Hz, 1H), 9.06 (d, *J* = 2.4 Hz, 1H); ^13^C NMR (75 MHz, DMSO-d_6_) δ 46.0, 55.3, 105.1, 113.9, 114.7, 127.0, 127.1, 128.2, 128.3, 128.3, 129.4, 131.4, 132.2, 133.3, 136.1, 143.3, 143.9, 145.6, 159.5, 164.2; HRMS (EI) for C_24_H_22_N_3_O_3_ (M + H^+^) calcd 400.1661, found 400.1659; HPLC purity of 99.02% (retention time = 32.49).

##### 4.1.2.8. 4-(((3–(2-Cyanophenyl)quinolin-8-yl)amino)methyl)-N-hydroxybenzamide amide (21)

The title compound was obtained in 38% overall yield from compound **39** following the general procedure for hydroxamic acid synthesis: ^1^H NMR (300 MHz, DMSO-d_6_) δ 4.68 (s, 2H), 4.68 (s, 2H), 6.65 (d, *J* = 7.5 Hz, 1H), 7.17 (d, *J* = 8.1 Hz, 1H), 7.35 (d, *J* = 8.1 Hz, 1H), 7.54 (d, *J* = 8.1 Hz, 2H), 7.63 (td, *J* = 0.9, 1.8 Hz, 7.5 Hz, 1H), 7.72 (d, *J* = 8.1 Hz, 2H), 7.75 (d, *J* = 7.5 Hz, 1H), 7.83 (d, *J* = 7.8 Hz, 1H), 7.93 (d, *J* = 7.5 Hz, 1H), 8.36 (d, *J* = 2.4 Hz, 1H), 8.91 (d, *J* = 2.1 Hz, 1H); ^13^C NMR (75 MHz, DMSO-d_6_) δ 52.0, 97.9, 103.0, 106.2, 109.9, 118.8, 118.9, 119.7, 120.1, 120.2, 122.1, 122.7, 123.4, 125.1, 125.5, 127.6, 129.3, 133.8, 135.7, 136.1, 138.1, 158.5; HRMS (EI) for C_24_H_19_N_4_O_2_ (M + H^+^) calcd 395.1508, found 395.1512; HPLC purity of 97.06% (retention time = 29.41).

##### 4.1.2.9. 4-(((3–(3-Cyanophenyl)quinolin-8-yl)amino)methyl)-N-hydroxybenzamide (22)

The title compound was obtained in 55% overall yield from compound **40** following the general procedure for hydroxamic acid synthesis: ^1^H NMR (300 MHz, DMSO-d_6_) δ 4.62 (d, *J* = 6.0 Hz, 2H), 6.56 (d, *J* = 7.8 Hz, 1H), 7.13 (d, *J* = 7.5 Hz, 1H), 7.30–7.35 (m, 2H), 7.47 (d, *J* = 8.1 Hz, 2H), 7.69 (d, *J* = 8.4 Hz, 2H), 7.76 (t, *J* = 8.1 Hz, 1H), 7.92 (d, *J* = 8.1 Hz, 1H), 8.25 (d, *J* = 8.4 Hz, 1H), 8.41 (d, *J* = 1.8 Hz, 1H), 8.62 (d, *J* = 2.1 Hz, 1H), 9.15 (d, *J* = 2.4 Hz, 1H); ^13 ^C NMR (75 MHz, DMSO-d_6_) δ 45.8, 105.5, 112.3, 113.9, 118.7, 126.9, 127.1, 128.0, 128.4, 130.3, 130.7, 131.2, 131.4, 131.6, 131.9, 133.8, 137.0, 138.5, 143.3, 144.1,145.5, 164.2; HRMS (EI) for C_24_H_18_N_4_O_2_ (M + H^+^) calcd 395.1508, found 395.1511; HPLC purity of 93.59% (retention time = 30.47).

##### 4.1.2.10. 4-(((3–(4-Cyanophenyl)quinolin-8-yl)amino)methyl)-N-hydroxybenzamide (23)

The title compound was obtained in 34% overall yield from compound **41** following the general procedure for hydroxamic acid synthesis: ^1^H NMR (300 MHz, DMSO-d_6_) δ 4.61 (d, *J* = 6.3 Hz, 2H), 6.57 (d, *J* = 7.2 Hz, 1H), 7.15 (d, *J* = 8.1 Hz, 1H), 7.33 (t, *J* = 7.8 Hz, 2H), 7.46 (d, *J* = 8.1 Hz, 2H), 7.69 (d, *J* = 8.1 Hz, 2H), 8.02 (d, *J* = 8.4 Hz, 2H), 8.12 (d, *J* = 8.4 Hz, 2H), 8.62 (d, *J* = 2.1 Hz, 1H), 9.14 (d, *J* = 2.1 Hz,1H); ^13 ^C NMR (75 MHz, DMSO-d_6_) δ 46.2, 106.1, 111.1, 114.5, 119.3, 127.4, 127.5, 128.4, 128.5, 128.9, 131.6, 133.5, 134.6, 137.5, 142.3, 143.7, 144.5, 146.0, 164.7; HRMS (EI) for C_24_H_19_N_4_O_2_ (M + H^+^) calcd 395.1508, found 395.1505; HPLC purity of 96.50% (retention time = 30.24).

##### 4.1.2.11. N-Hydroxy-4-(((3–(3-nitrophenyl)quinolin-8-yl)amino)methyl)benzamide (24)

The title compound was obtained in 50% overall yield from compound **42** following the general procedure for hydroxamic acid synthesis: ^1^H NMR (300 MHz, DMSO-d_6_) δ 4.62 (d, *J* = 6.0 Hz, 2H), 6.57 (d, *J* = 7.8 Hz, 1H), 7.18 (d, *J* = 7.5 Hz, 1H), 7.31–7.36 (m, 2H), 7.47 (d, *J* = 7.8 Hz, 2H), 7.69 (d, *J* = 8.1 Hz, 2H), 7.85 (t, *J* = 7.8 Hz, 1H), 8.31 (d, *J* = 7.5 Hz, 1H), 8.37 (d, *J* = 8.1 Hz, 1H), 8.67 (s, 2H), 9.17 (d, *J* = 1.8 Hz, 1H); ^13 ^C NMR (75 MHz, DMSO-d_6_) δ 46.3, 106.0, 114.5, 122.1, 123.2, 127.4, 127.5, 128.4, 128.9, 131.2, 131.5, 131.9, 134.2, 134.5, 137.5, 139.5, 143.7, 144.6, 146.0,149.1; HRMS (EI) for C_23_H_19_N_4_O_4_ (M + H^+^) calcd 415.1406, found 415.1408; HPLC purity of 93.65% (retention time = 32.15).

##### 4.1.2.12. N-Hydroxy-4-(((3-(pyridin-3-yl)quinolin-8-yl)amino)methyl)benzamide (25)

The title compound was obtained in 41% overall yield from compound **43** following the general procedure for hydroxamic acid synthesis: ^1^H NMR (300 MHz, DMSO-d_6_) δ 4.62 (d, *J* = 6.3 Hz, 2H), 6.56 (t, *J* = 7.5 Hz, 1H), 7.14 (d, *J* = 7.5 Hz, 1H), 7.32 (d, *J* = 8.1 Hz, 2H), 7.47 (d, *J* = 8.4 Hz, 2H), 7.58 (dd, *J* = 4.5 Hz, 7.9 Hz, 1H), 7.69 (d, *J* = 8.1 Hz, 2H), 8.30 (td, *J* = 1.8 Hz, 4.2 Hz, 1H), 8.59 (d, *J* = 2.4 Hz, 1H), 8.66 (dd, *J* = 1.8 Hz, 4.8 Hz, 1H), 9.10 (d, *J* = 1.8 Hz, 1H), 9.14 (d, *J* = 2.4 Hz, 1H); ^13 ^C NMR (75 MHz, DMSO-d_6_) δ 45.7, 105.3, 113.7, 124.0, 126.8, 127.0, 127.9, 128.2, 130.2, 131.3, 132.8, 133.5, 134.5, 136.8, 143.2, 144.0, 145.5, 147.9, 149.0, 164.1; HRMS (EI) for C_22_H_19_N_4_O_2_ (M + H^+^) calcd 371.1508, found 371.1507; HPLC purity of 98.11% (retention time = 19.77).

##### 4.1.2.13. N-Hydroxy-4-(((3-(pyridin-4-yl)quinolin-8-yl)amino)methyl)benzamide (26)

The title compound was obtained in 40% overall yield from compound **44** following the general procedure for hydroxamic acid synthesis: ^1^H NMR (300 MHz, DMSO-d_6_) δ 4.63 (s, 2H), 6.62 (d, *J* = 7.5 Hz, 1H), 7.19 (d, *J* = 7.8 Hz, 1H), 7.37 (t, *J* = 8.1 Hz, 1H), 7.47 (d, *J* = 8.1 Hz, 2H), 7.69(d, *J* = 8.1 Hz, 2H), 8.27 (d, *J* = 6.0 Hz, 2H), 8.81 (d, *J* = 2.1 Hz, 1H), 8.89 (d, *J* = 5.7 Hz, 2H), 9.26 (d, *J* = 2.1 Hz, 1H); ^13 ^C NMR (75 MHz, DMSO-d_6_) δ 45.8, 106.5, 114.2, 123.1, 126.9, 127.0, 127.7, 128.8, 131.4, 135.4, 137.7, 143.2, 144.1, 145.2, 145.8, 149.5, 164.2, 179.2; HRMS (EI) for C_22_H_19_N_4_O_2_ (M + H^+^) calcd 371.1508, found 371.1505; HPLC purity of 94.78% (retention time = 16.94).

##### 4.1.2.14. 4-(((3-(Furan-2-yl)quinolin-8-yl)amino)methyl)-N-hydroxybenzamide (27)

The title compound was obtained in 32% overall yield from compound **45** following the general procedure for hydroxamic acid synthesis: ^1^H NMR (300 MHz, DMSO-d_6_) δ 4.59 (d, *J* = 6.3 Hz, 2H), 6.51 (d, *J* = 6.9 Hz, 1H), 6.70 (dd, *J* = 1.8 Hz, 3.4 Hz, 1H), 7.11 (d, *J* = 8.1 Hz, 1H), 7.25 (d, *J* = 4.2 Hz, 2H), 7.29 (d, *J* = 8.1 Hz, 1H), 7.46 (d, *J* = 8.1 Hz, 2H), 7.69 (d, *J* = 8.1 Hz, 2H), 7.88 (dd, *J* = 0.6 Hz, 1.8 Hz, 1H), 8.43 (d, *J* = 2.4 Hz, 1H), 9.14 (d, *J* = 2.4 Hz, 1H); ^13 ^C NMR (75 MHz, DMSO-d_6_) δ 45.8, 105.1, 107.5, 112.3, 113.7, 123.9, 126.8, 126.9, 127.9, 128.4, 128.8, 131.4, 136.2, 143.1, 143.8, 144.1, 150.7, 164.0; HRMS (EI) for C_21_H_18_N_3_O_3_ (M + H^+^) calcd 360.1348, found 360.1350; HPLC purity of 96.16% (retention time = 30.57).

##### 4.1.2.15. 4-(((3-(Furan-3-yl)quinolin-8-yl)amino)methyl)-N-hydroxybenzamide (28)

The title compound was obtained in 32% overall yield from compound **46** following the general procedure for hydroxamic acid synthesis: ^1^H NMR (300 MHz, DMSO-d_6_) δ 4.59 (d, *J* = 6.3 Hz, 2H), 6.49 (d, *J* = 8.1 Hz, 1H), 7.04 (d, *J* = 7.5 Hz, 1H), 7.18 (s, 1H), 7.23 (d, *J* = 6.9 Hz, 1H), 7.28 (d, *J* = 7.8 Hz, 1H), 7.46 (d, *J* = 8.1 Hz, 2H), 7.69 (d, *J* = 8.4 Hz, 2H), 7.84 (s, 1H), 8.39 (d, *J* = 2.1 Hz, 1H), 8.45 (s, 1H), 9.07 (d, *J* = 2.1 Hz, 1H);^13^C NMR (75 MHz, DMSO-d_6_) δ 45.8, 104.7, 108.6, 113.4, 123.0, 125.6, 126.8, 127.0, 128.1, 128.2, 130.9, 131.3, 136.3, 140.2, 143.4, 144.1, 144.6, 145.2, 164.2; HRMS (EI) for C_21_H_18_N_3_O_3_ (M + H^+^) calcd 360.1348, found 360.1348; HPLC purity of 98.61% (retention time = 29.15).

##### 4.1.2.16. N-Hydroxy-4-(((3-(thiophen-3-yl)quinolin-8-yl)amino)methyl)benzamide (29)

The title compound was obtained in 28% overall yield from compound **47** following the general procedure for hydroxamic acid synthesis: ^1^H NMR (300 MHz, DMSO-d_6_) δ 4.60 (s, 2H), 6.50 (dd, *J* = 0.9 Hz, 7.8 Hz, 1H), 7.08 (dd, *J* = 0.9 Hz, 8.1 Hz, 1H), 7.28 (t, *J* = 7.8 Hz, 1H), 7.47 (d, *J* = 8.1 Hz, 2H), 7.69 (d, *J* = 8.4 Hz, 2H), 7.74–7.80 (m, 2H), 8.18 (dd, *J* = 1.5 Hz, 2.7 Hz, 1H), 8.51 (d, *J* = 2.4 Hz, 1H), 9.17 (d, *J* = 2.1 Hz, 1H); ^13 ^C NMR (75 MHz, DMSO-d_6_) δ 45.9, 105.0, 113.7, 122.4, 126.3, 126.9, 127.1, 127.6, 128.2, 128.2, 128.5, 131.4, 131.8, 136.3, 138.4, 143.4, 144.1, 145.6, 164.2; HRMS (EI) for C_21_H_18_N_3_O_2_S (M + H^+^) calcd 376.1120, found 376.1122; HPLC purity of 97.78% (retention time = 31.49).

### 4.2. Biology

#### 4.2.1. Tumour cell culture

All human cancer cells were maintained in RPMI 1640 medium supplemented with 10% FBS and penicillin (100 units/ml)/streptomycin (100 µg/ml)/amphotericin B (0.25 µg/ml). All cells were maintained in humidified air containing 5% CO_2_ at 37 °C and cultured every 2–3 days. All cells were cultured in tissue culture flasks in humidified air containing 5% CO_2_ at 37 °C and cultured every 2–3 days.

#### 4.2.2. The sulforhodamine B assays

Cells were seeded at the density of 5000 cells/well into 96-plate overnight. Basal cells were fixed with 10% trichloroacetic acid (TCA) to represent the cell population at the time of compound addition (T_0_). After additional incubation of DMSO (C) or different doses of test compounds (Tx) for 48 h, cells were fixed with 10% TCA and stained with SRB at 0.4% (w/v) in 1% AcOH. Unbound SRB was washed out using 1% AcOH and SRB bound cells were solubilised with 10 mM Trizma base. The absorbance was read at a wavelength of 515 nm. The 50% growth inhibition (GI_50_) was calculated by 100 − [(T_x_ – T_0_)/(C – T_0_)] × 100.

#### 4.2.3. HDAC biochemical assays

The HDACs *in vitro* activities of human recombinant HDAC 1, 2, 4, 6, and 8 were conducted by EurofinPanlabs (Taipei, Taiwan). In brief, indicated compounds were incubated with specific HDAC enzyme and Fluor-de-Lys deacetylase substrate. Fluor-de-Lys deacetyl substrates were spectrofluorimetrically quantitated compared to control.

#### 4.2.4. Western blot analysis

Cells were incubated with indicated compounds for 24 h and lysed with ice-cold lysis buffer (20 mMTris-HCl pH 7.5, 150 mM NaCl, 1 mM Na_2_EDTA, 1 mM EGTA, 1% NP-40, 1% sodium deoxycholate, 0.1% SDS, 2.5 mM β-glycerylphosphate, 1 mM Na_4_P_2_O_7_, 5 mM NaF, 1 mM Na_3_VO_4_ and protease inhibitor cocktail from Millipore) on ice for 30 min following by centrifugation at 13000 rpm for 30 min. Protein concentrations were determined and equal amounts of protein were separated by 8–15% sodium dodecyl sulphate-polyacrylamide gel electrophoresis (SDS–PAGE) and transferred to poly(vinylidene difluoride) (PVDF) membranes. Membranes were immunoblotted with specific antibodies overnight at 4 °C and then applied to appropriate horseradish peroxidase-conjugated anti-mouse or anti-rabbit IgG secondary antibodies for 1 h at RT. Signals were detected using an enhanced chemiluminescence (Amersham, Buckinghamshire, UK).

#### 4.2.5. Flow cytometry

Briefly, A549 cells were starved with EBM-2 medium overnight and were subsequently replenished with EGM-2 medium with or without **17**, **25**, and **26** (0.25, 0.5, 10 µM) for 48 h. After being trypsinized and fixed in ice-cold 75% MeOH for 1 h at −20 °C, A549 cells were washed with PBS and resuspended in 0.2 ml DNA extraction buffer (0.2 M Na_2_HPO_4_, 0.1 M citric acid; pH 7.8) for 30 min. Then the cells were stained with propidium iodide solution (PI; 100 µg/ml RNase, 80 µg/ml propidium iodide, 0.1% Triton X-100) in PBS. FACScan flow cytometry was utilised to determine cell cycle distribution, and data analysis was performed with CellQuest software (BD Biosciences).

#### 4.2.8. Statistical and graphical analyses

Each experiment was performed independently at least three times and the data are presented as mean ± SEM for the indicated number of separate experiments. Student’s *t*-test was used to compare the mean of each group with that of the control group in experiments and one-way ANOVA was used in animal study. *p* Values <0.05 were considered significant (*p < 0.05, **p < 0.01, ***p < 0.001).

## Supplementary Material

Supplemental Material
