# Supplementary material for: Effect of 3-subsitution of quinolinehydroxamic acids on selectivity of histone deacetylase isoforms
Source: J Enzyme Inhib Med Chem. 2020 Nov 8;36(1):74–84. doi: 10.1080/14756366.2020.1839446 (PMC7655065; doi:10.1080/14756366.2020.1839446)

Support Information

Effect of 3-Subsitution of Quinolinehydroxamic Acids on Selectivity of Histone Deacetylase Isoforms

Samir Mehndiratta, Mei-Chuan Chen, Yuh-Hsuan Chao, Cheng-Hsin Lee, Jing-Ping Liou, Mei-Jung Lai,* Hsueh-Yun Lee*

Contents:

^{1}H NMR Spectra for compounds **14-29**……………………………………………SI-2
^{13}C NMR Spectra for compounds **14-29**…………………………………………SI-18

[1]H Spectra for compound 14

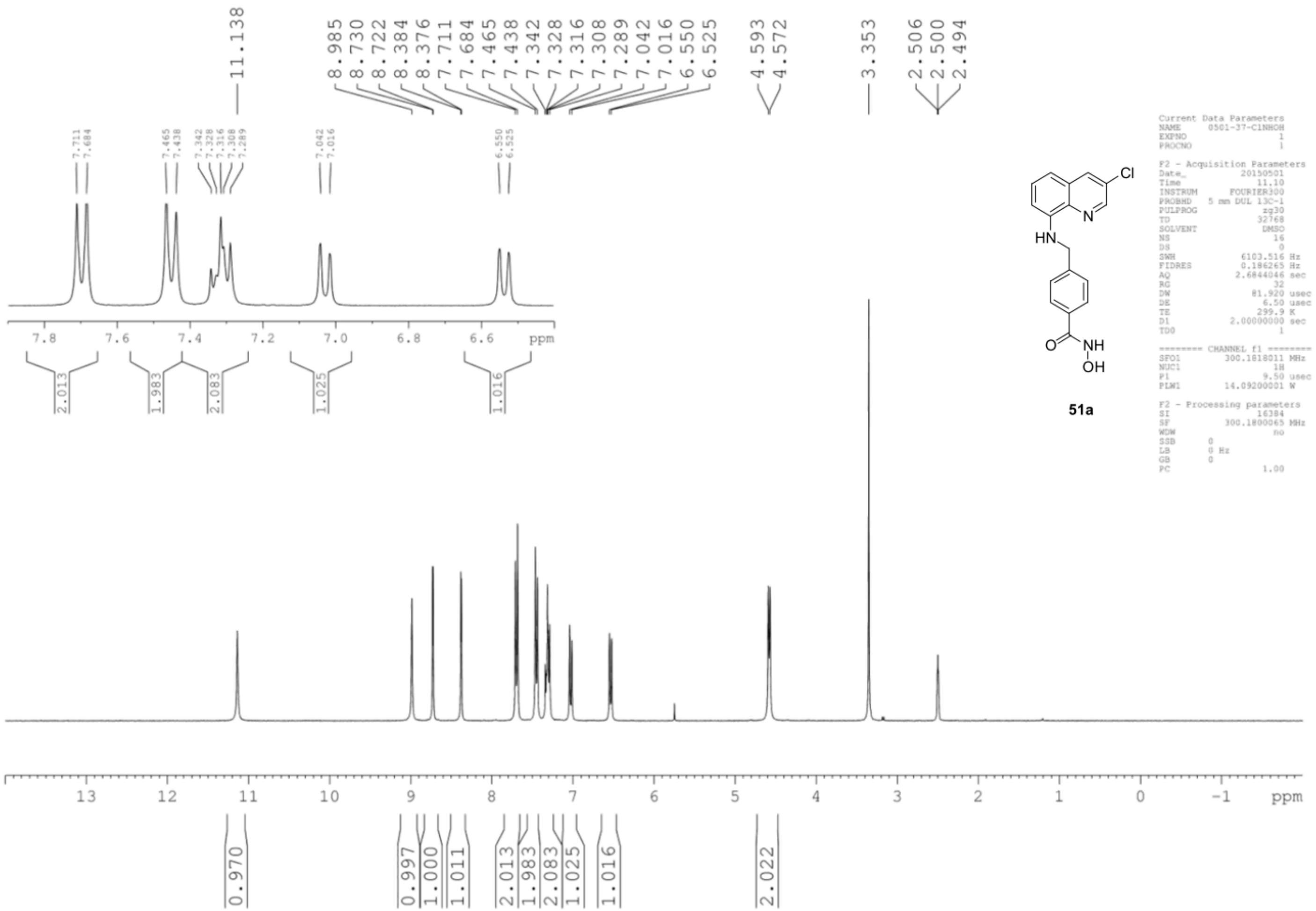

[1]H Spectra for compound 15

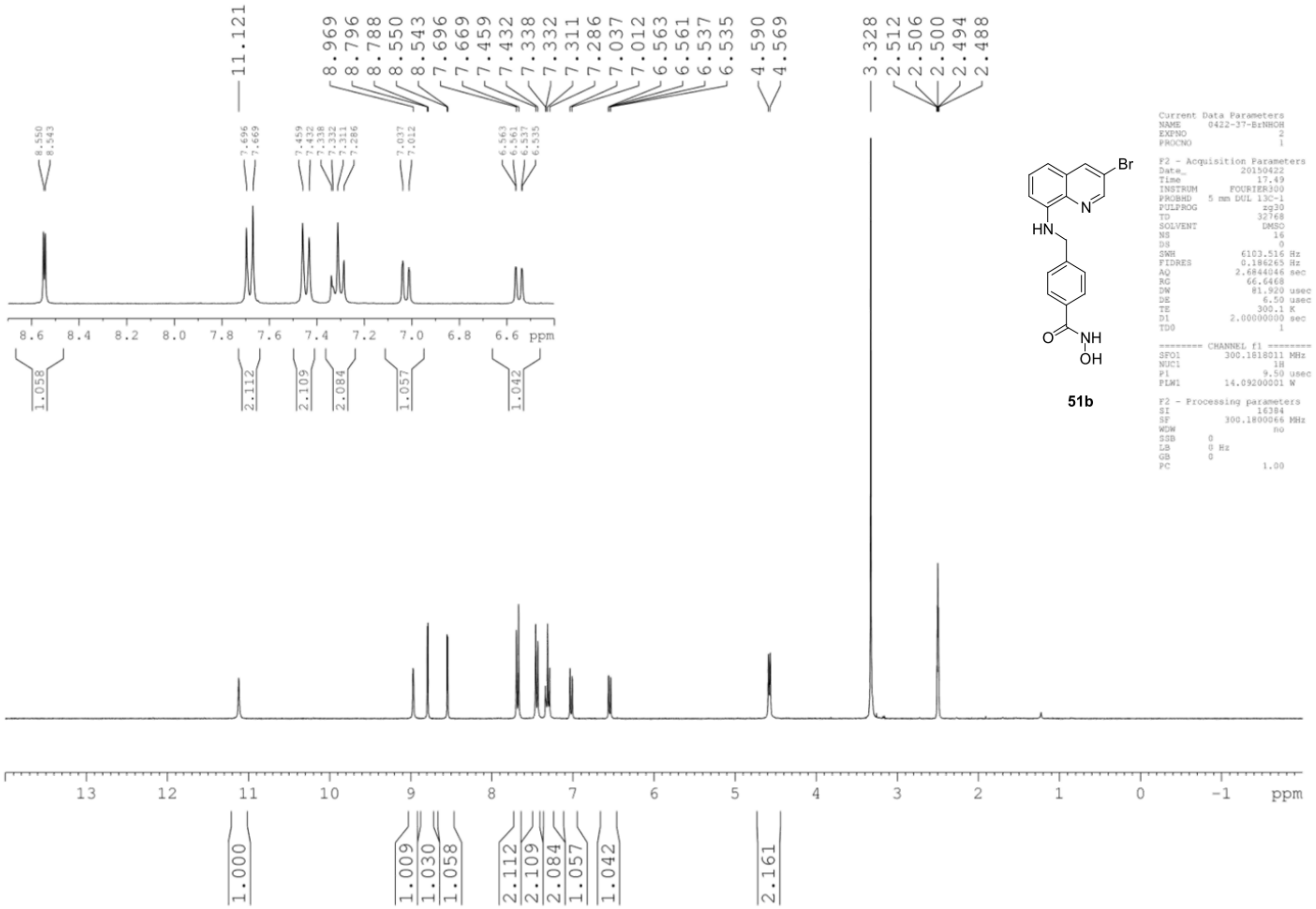

[1]H Spectra for compound 16

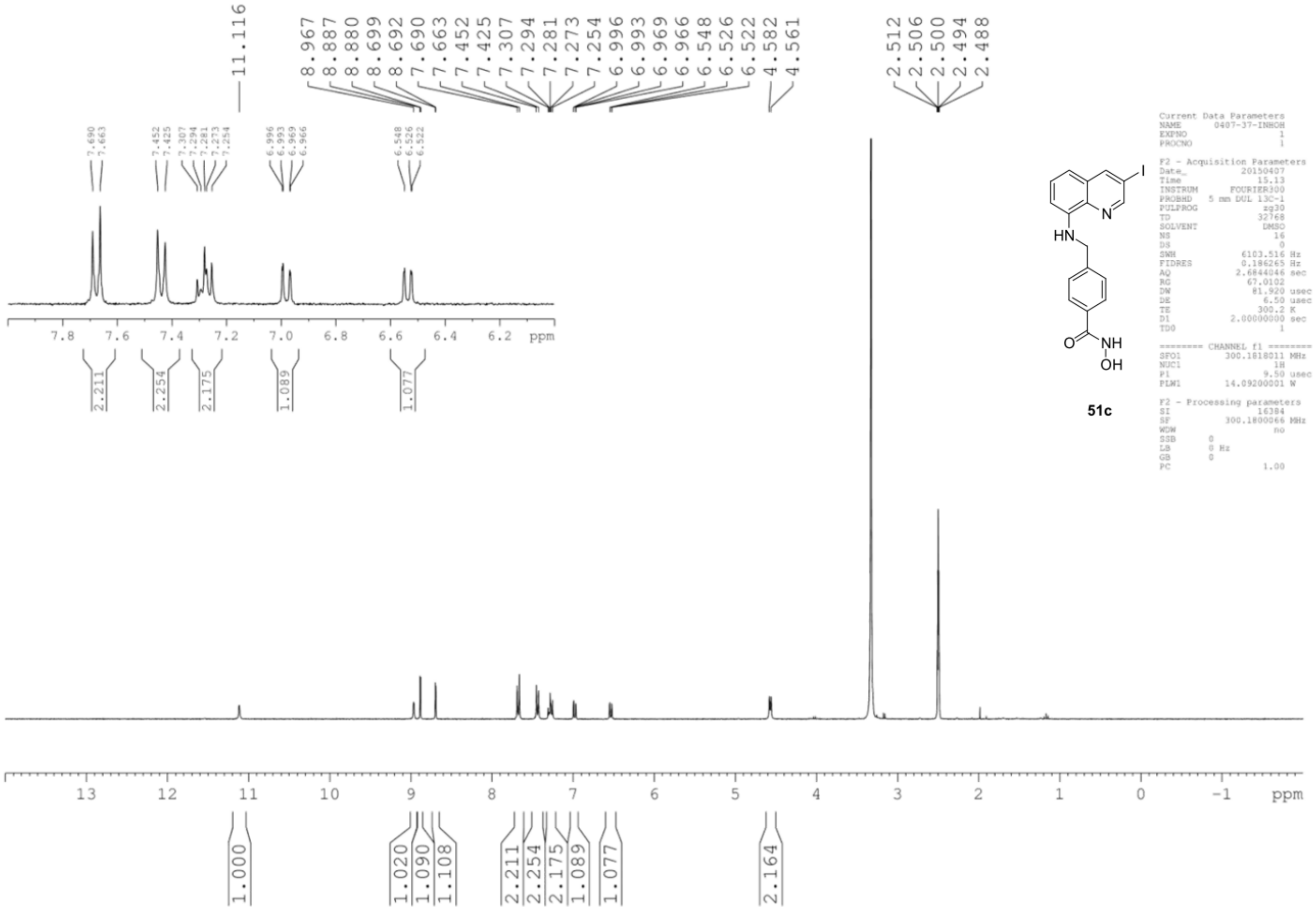

[1]H Spectra for compound 17

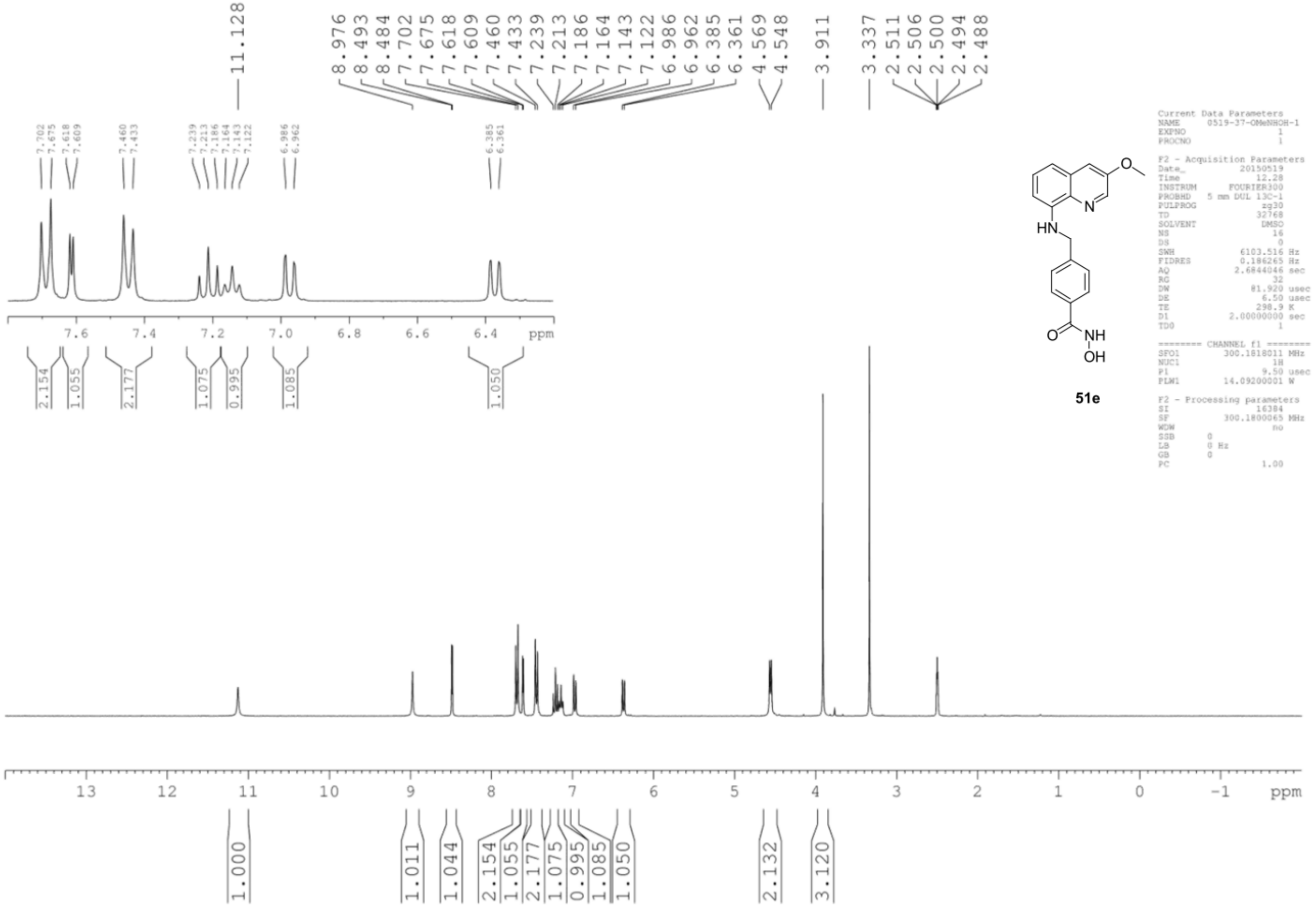

51e

^{1}H Spectra for compound 18

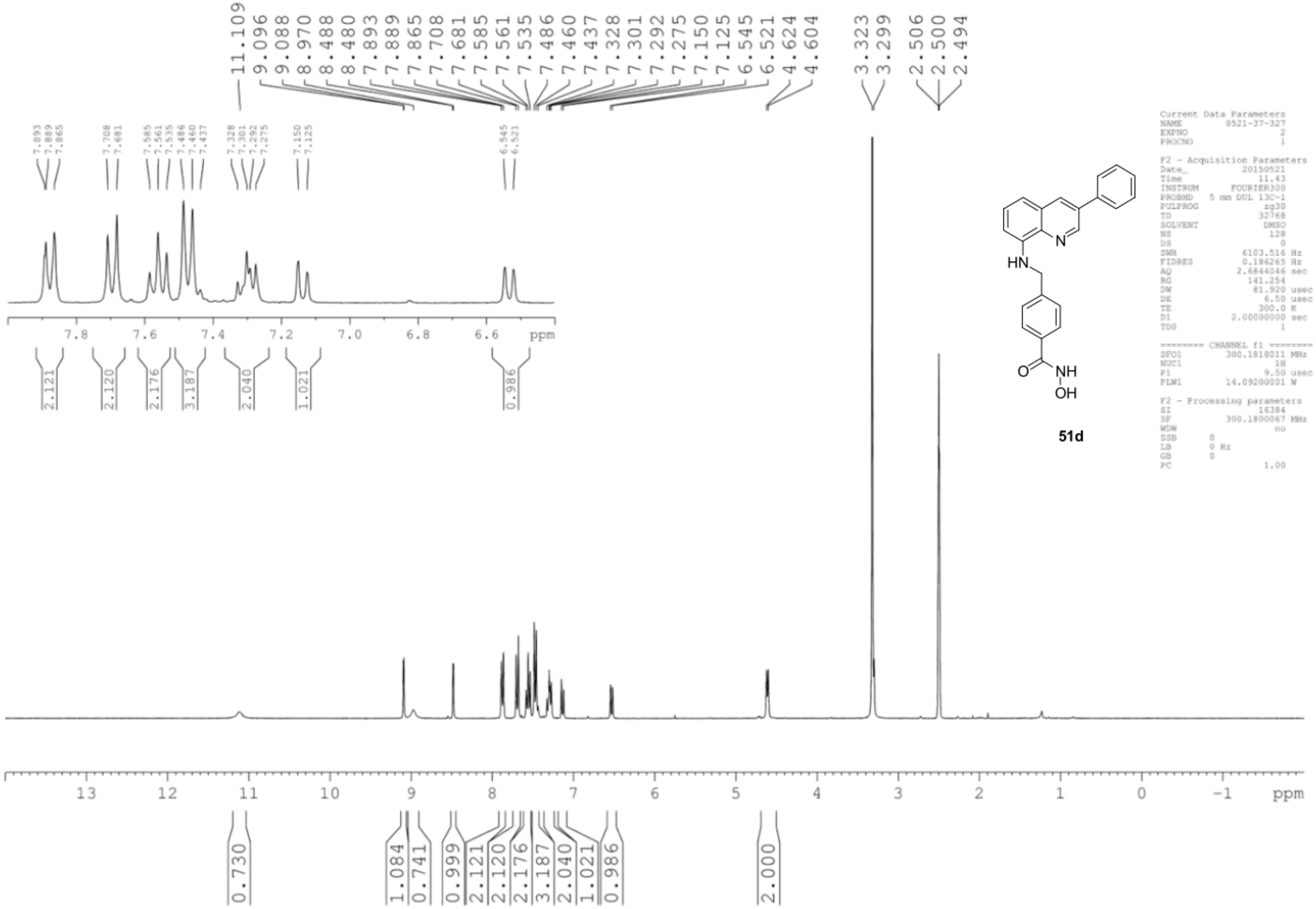

[1]H Spectra for compound 19

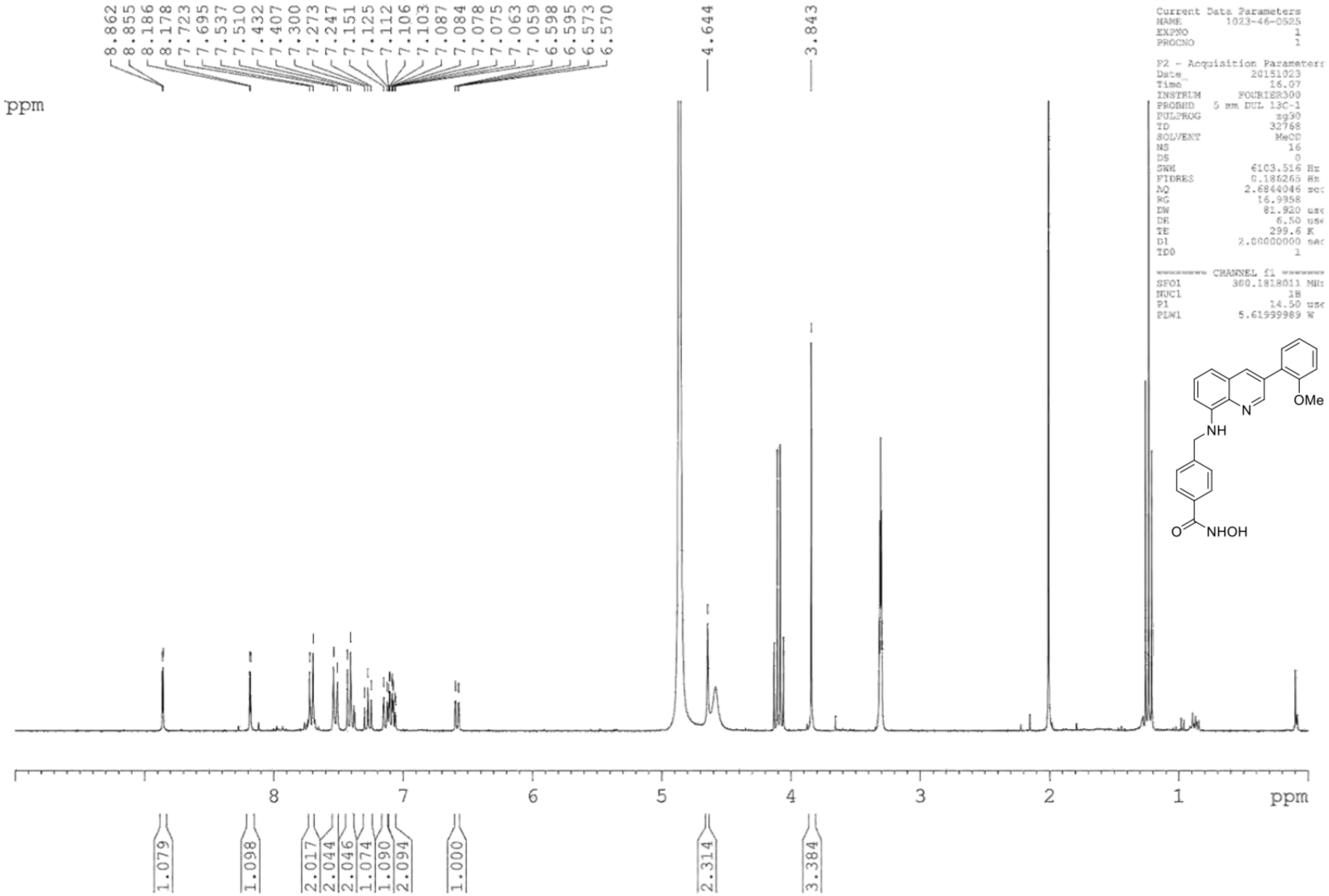

[1]H Spectra for compound 20

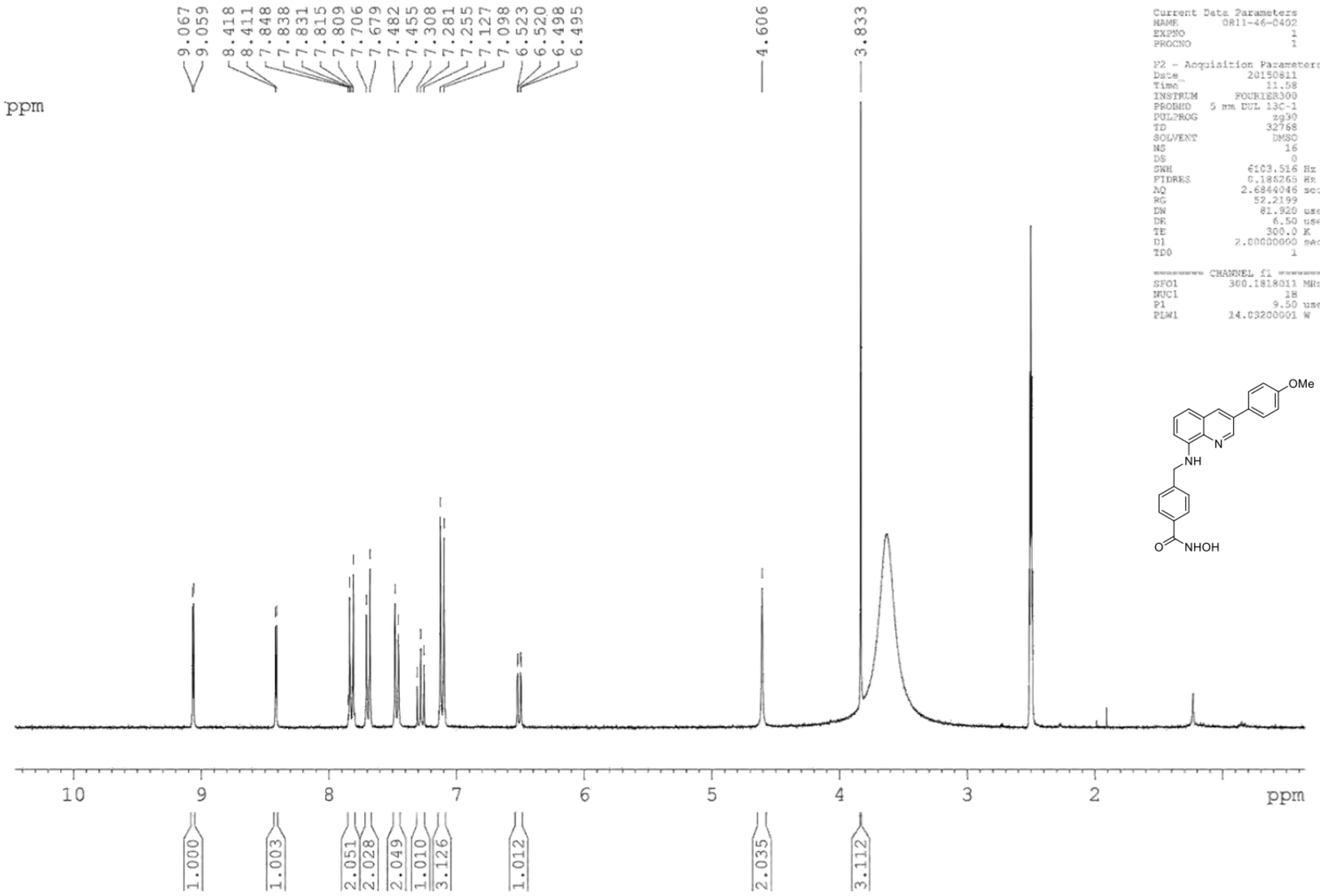

[1]H Spectra for compound 21

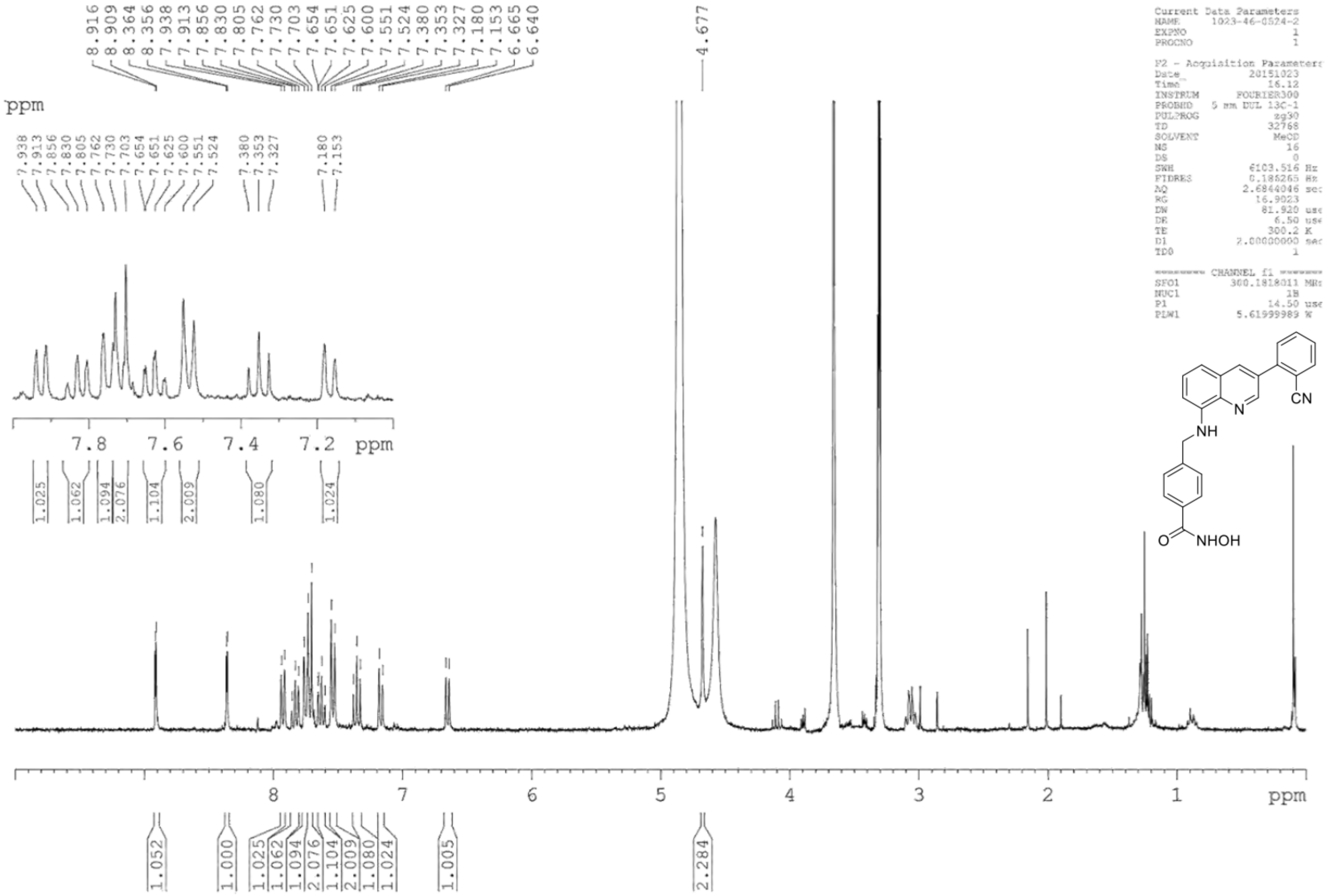

[1]H Spectra for compound 22

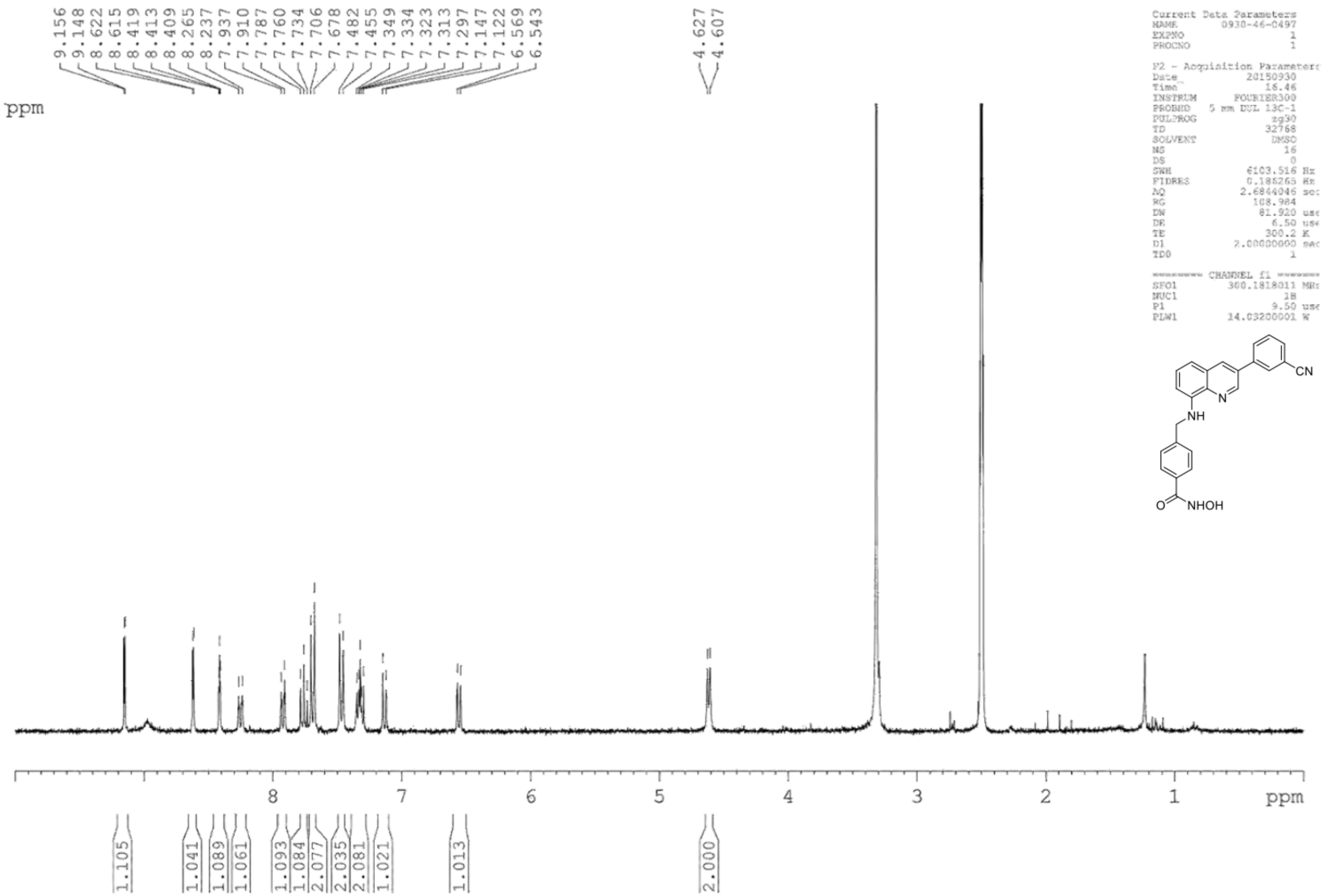

[1]H Spectra for compound 23

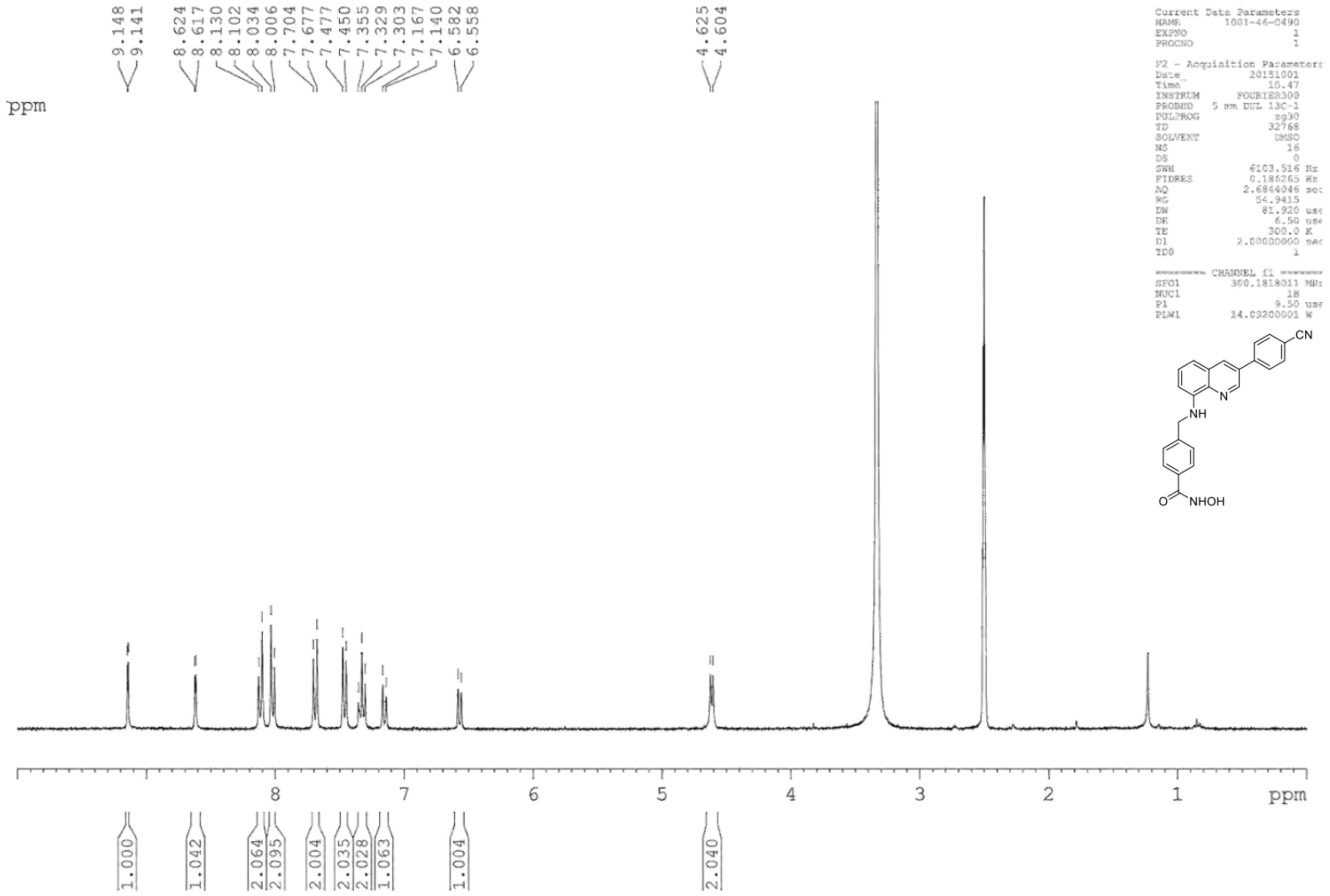

[1]H Spectra for compound 24

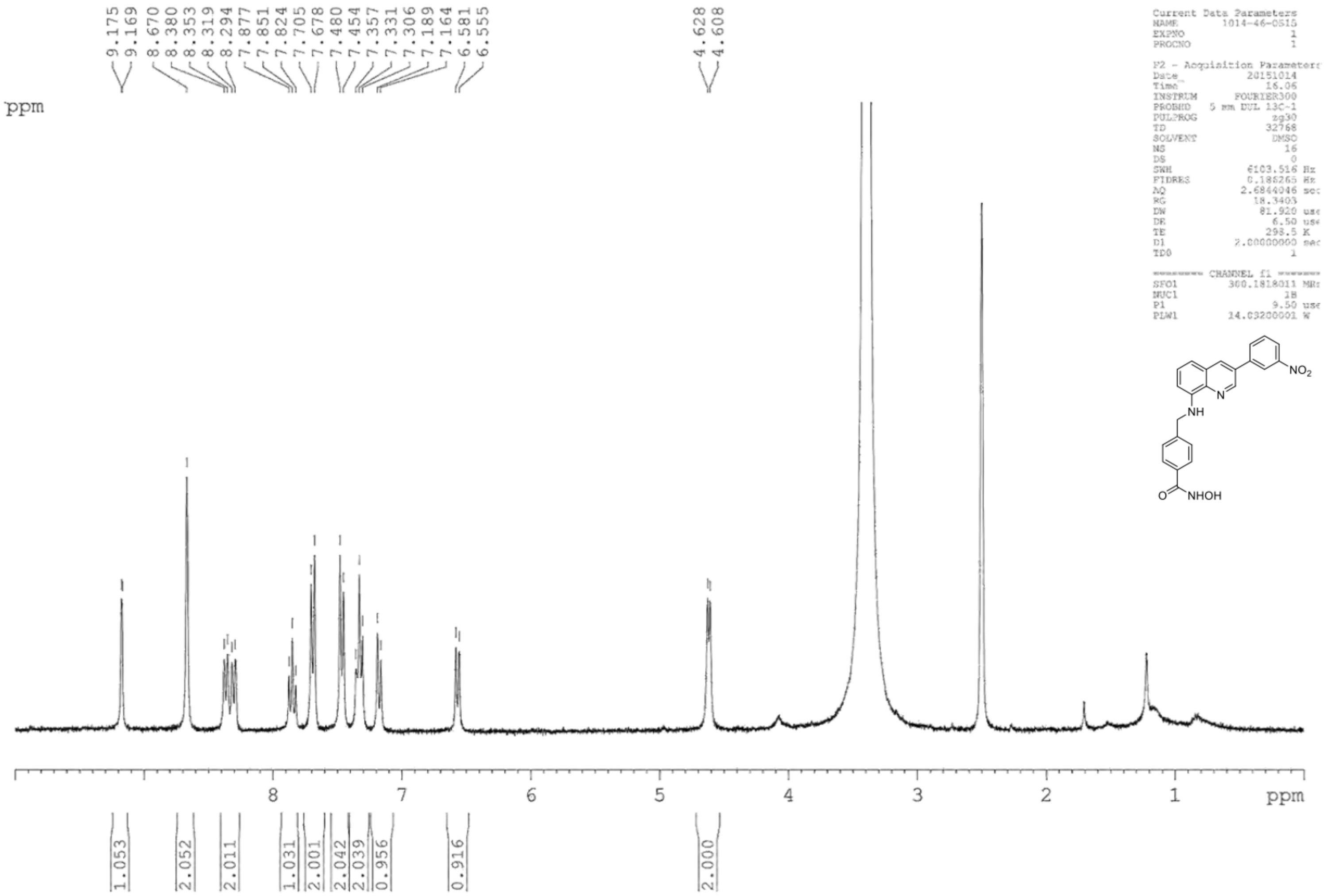

[1]H Spectra for compound 25

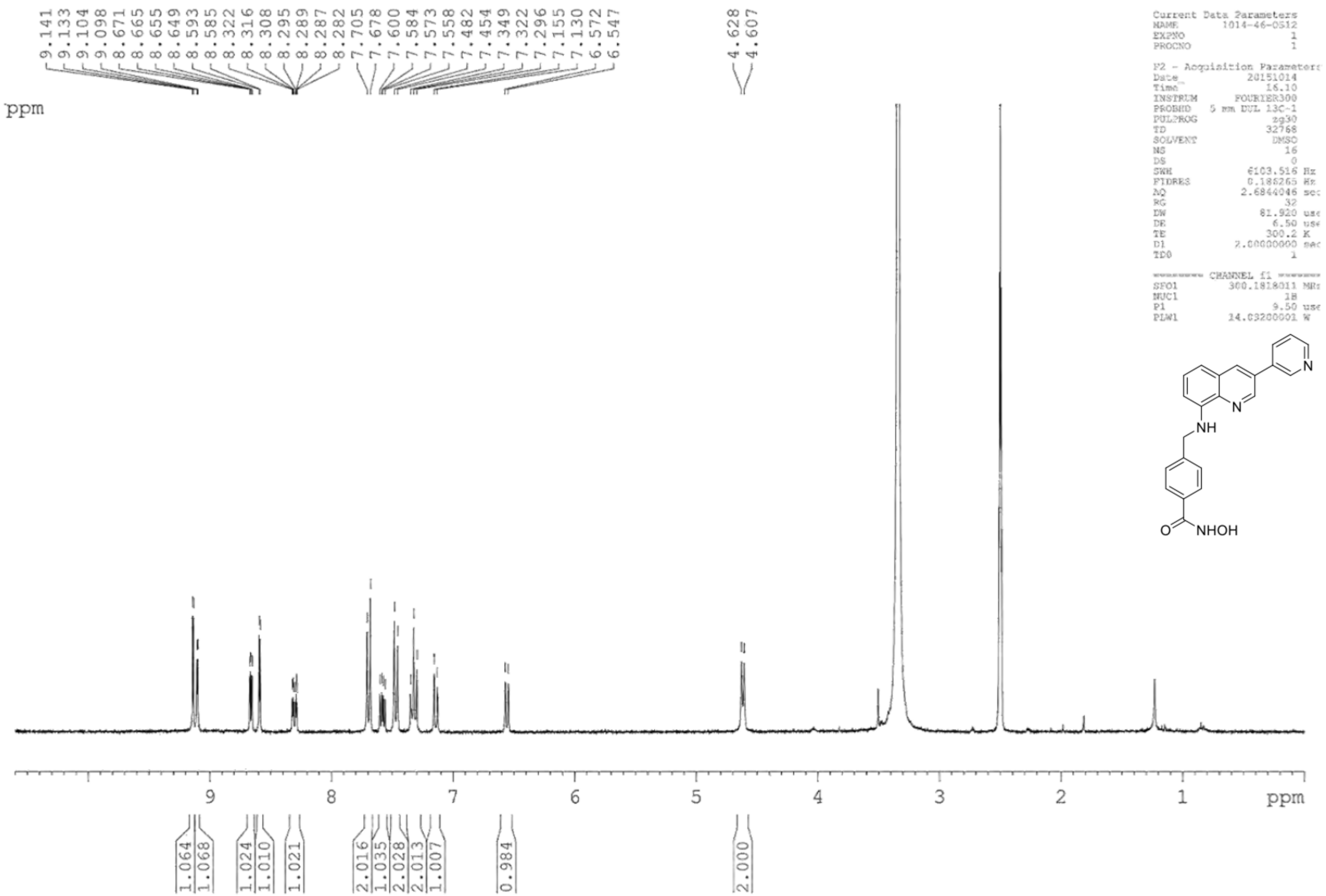

[1]H Spectra for compound 26

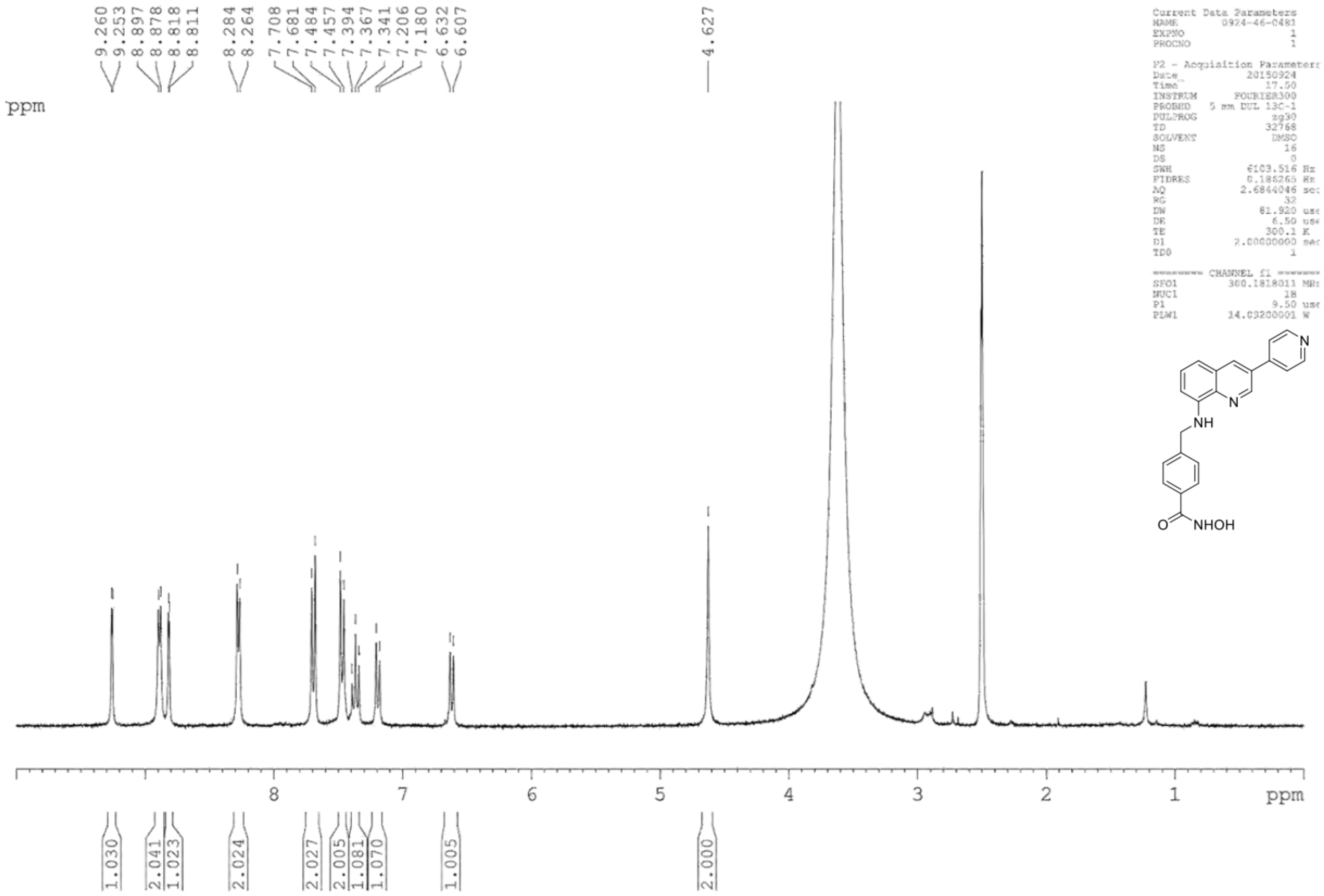

^{1}H Spectra for compound 27

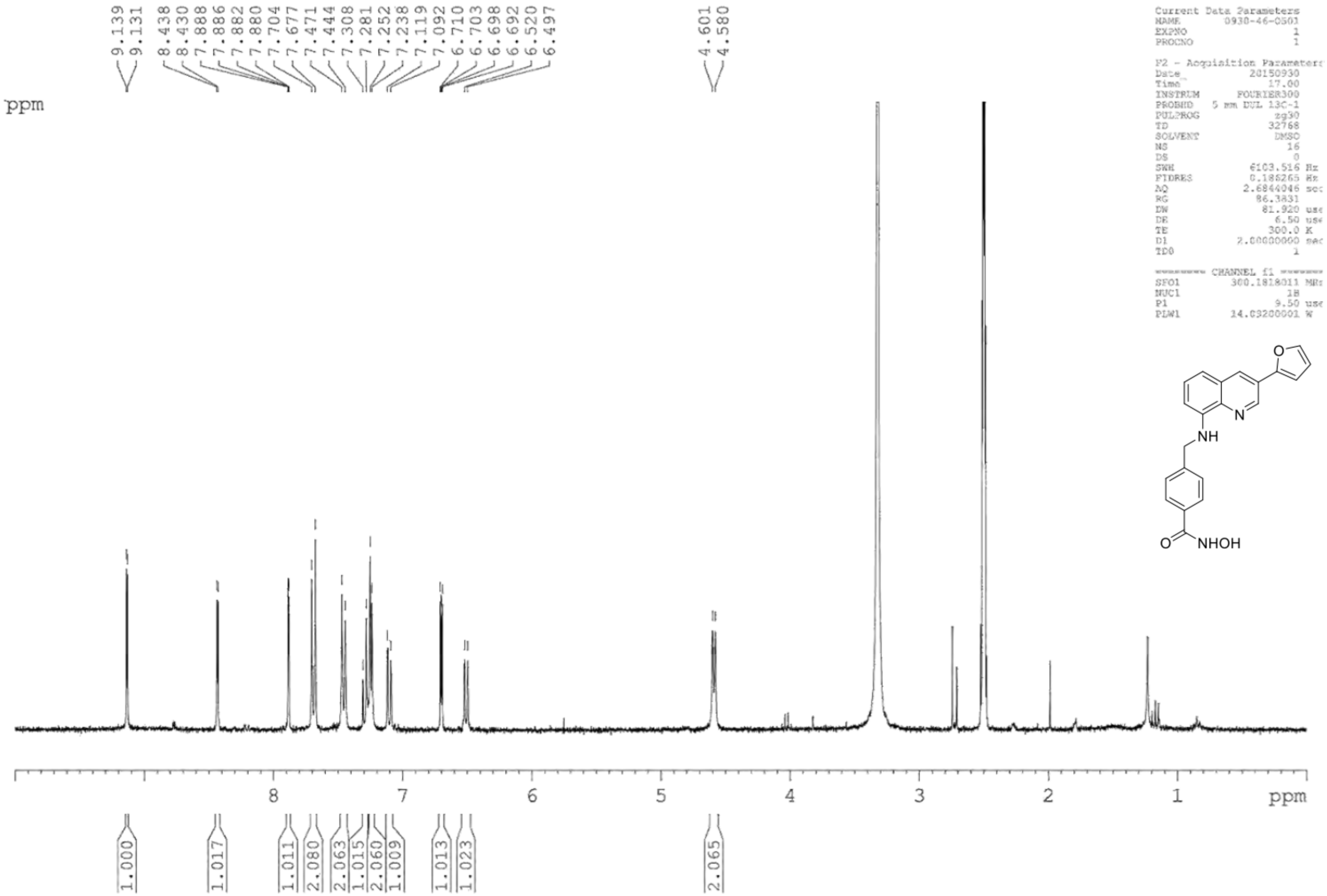

[1]H Spectra for compound 28

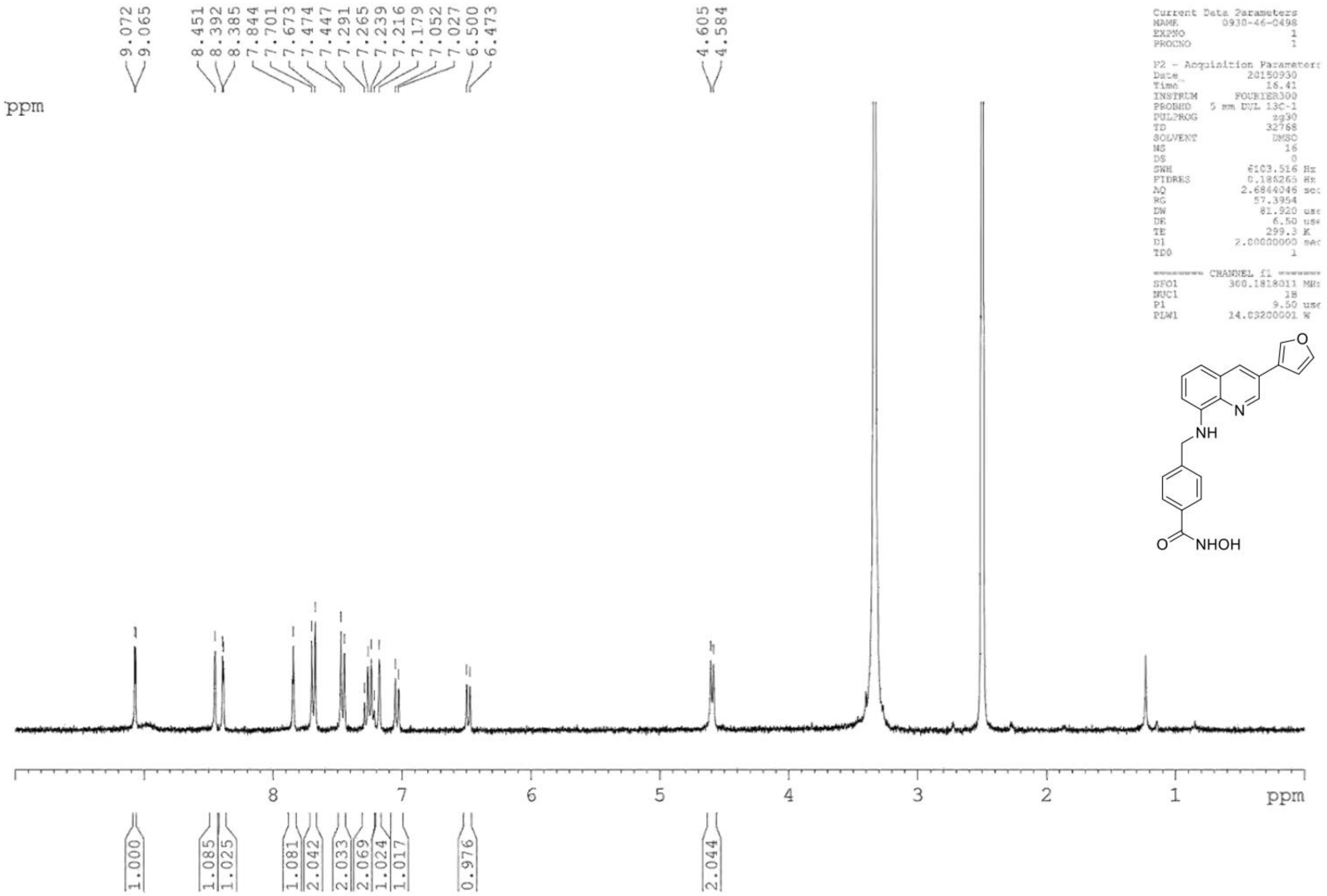

[1]H Spectra for compound 29

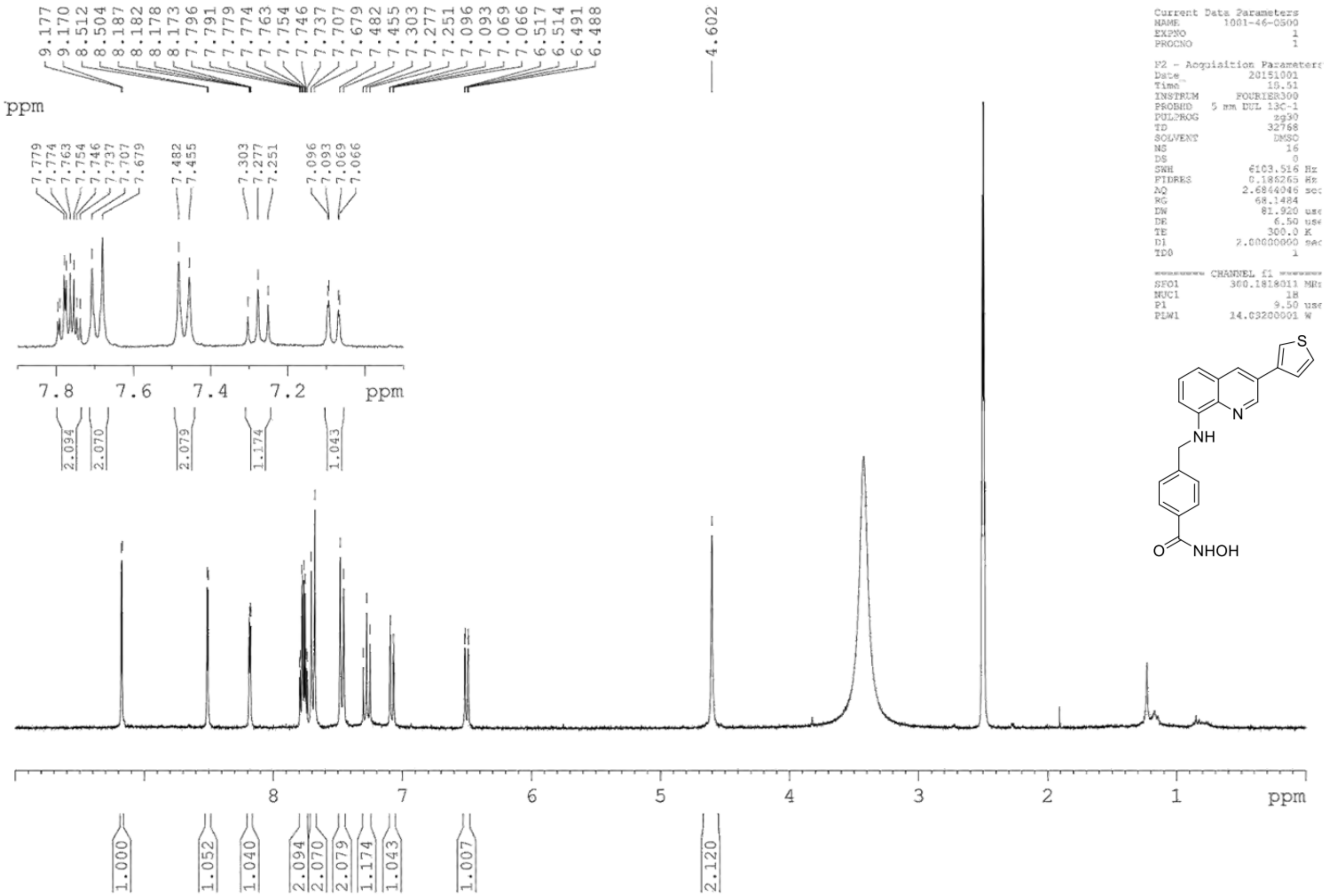

^{13}C Spectra for compound 14

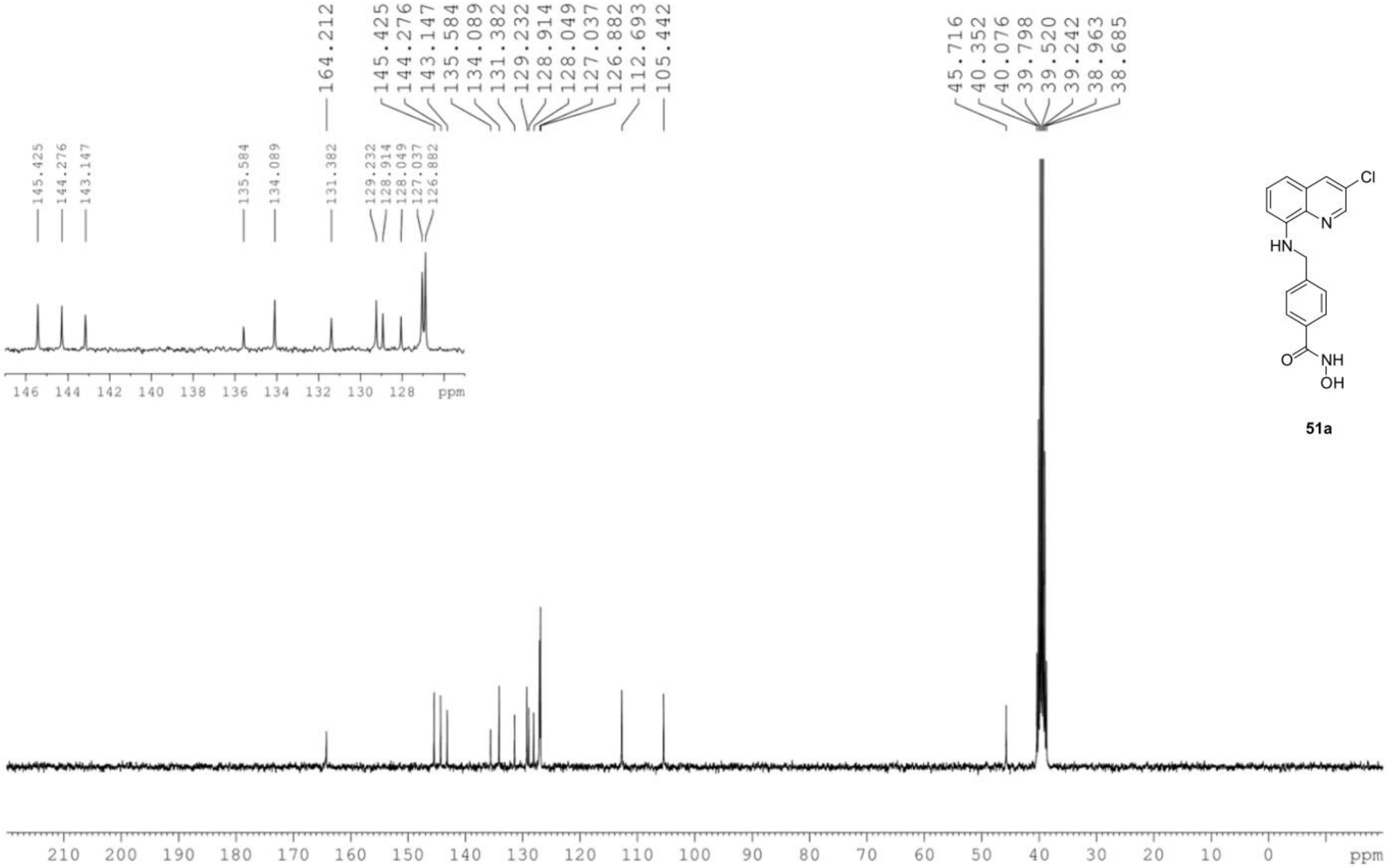

51a

^{13}C Spectra for compound 15

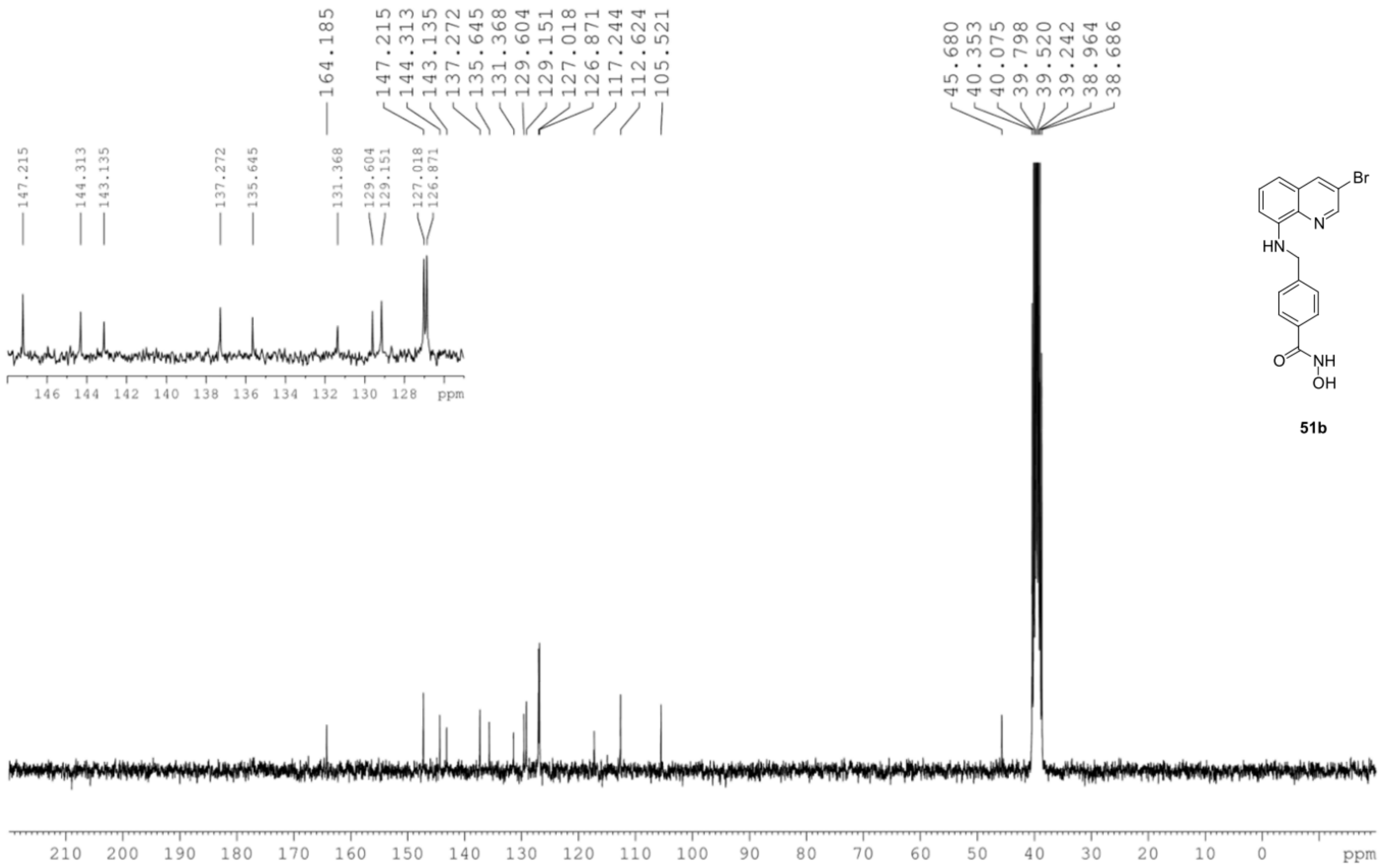

51b

^{13}C Spectra for compound 16

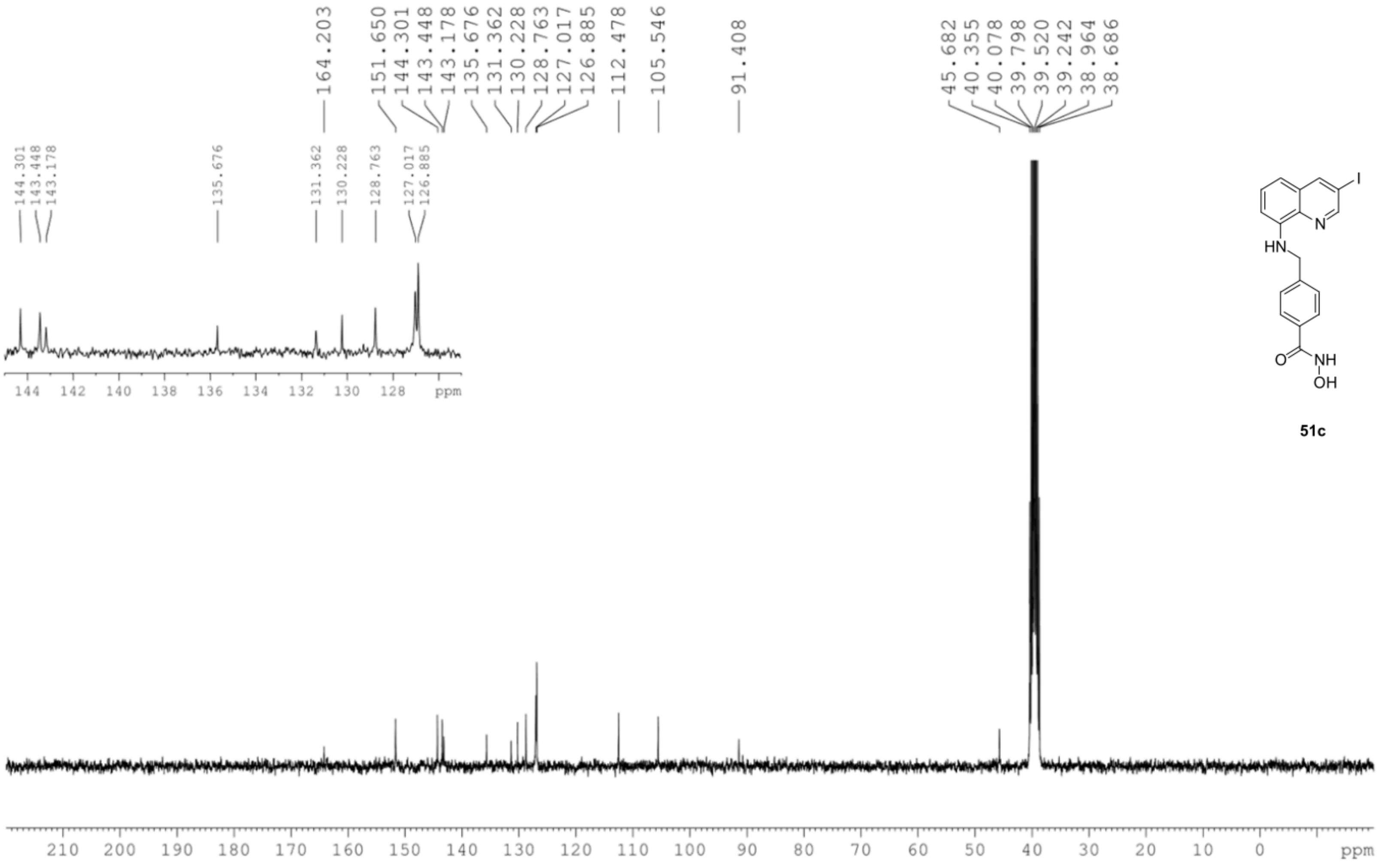

51c

^{13}C Spectra for compound 17

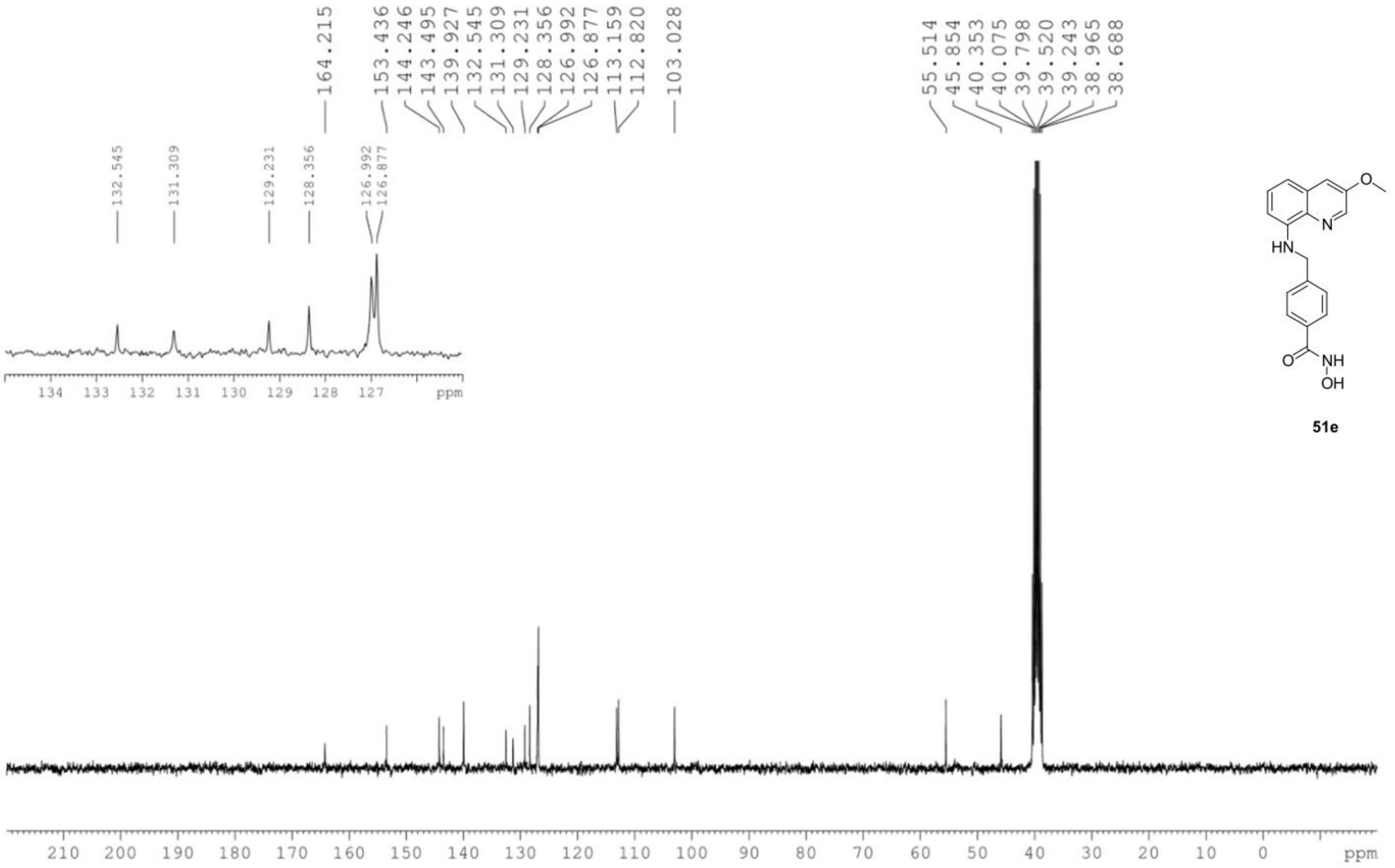

51e

^{13}C Spectra for compound 18

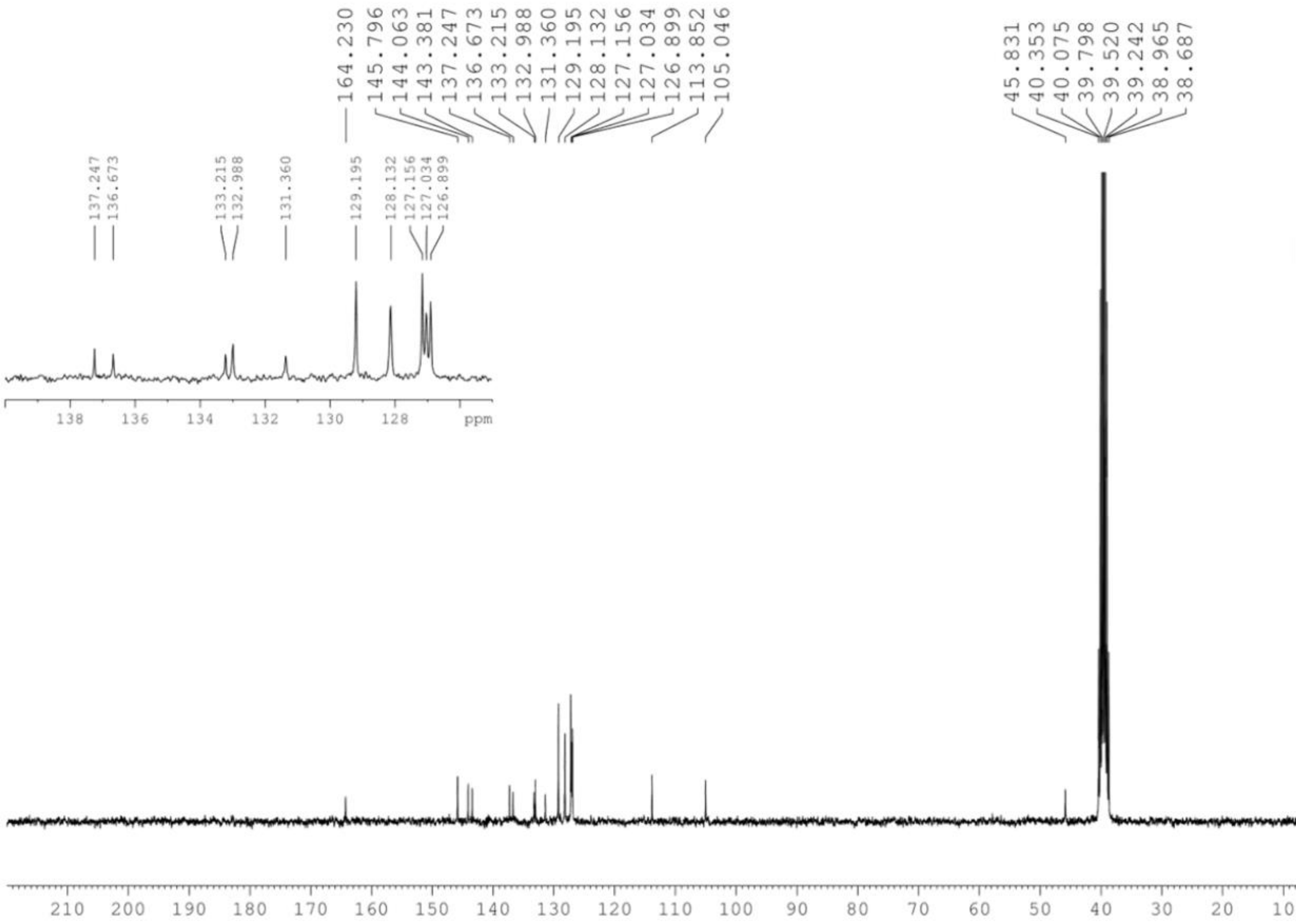

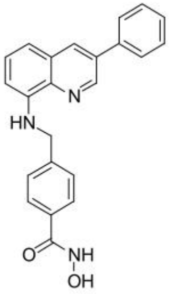

51d

[13]C Spectra for compound 19

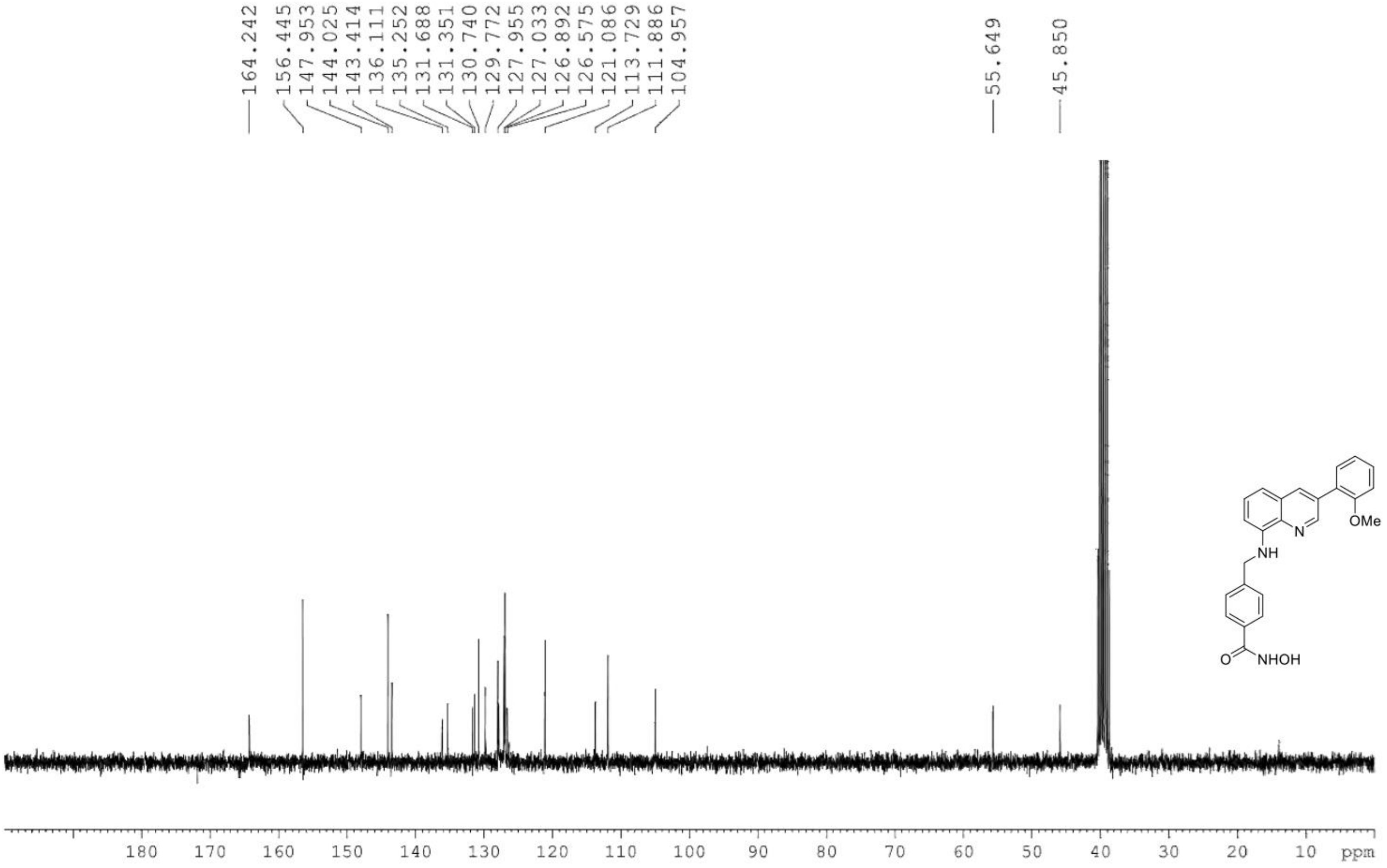

[13]C Spectra for compound 20

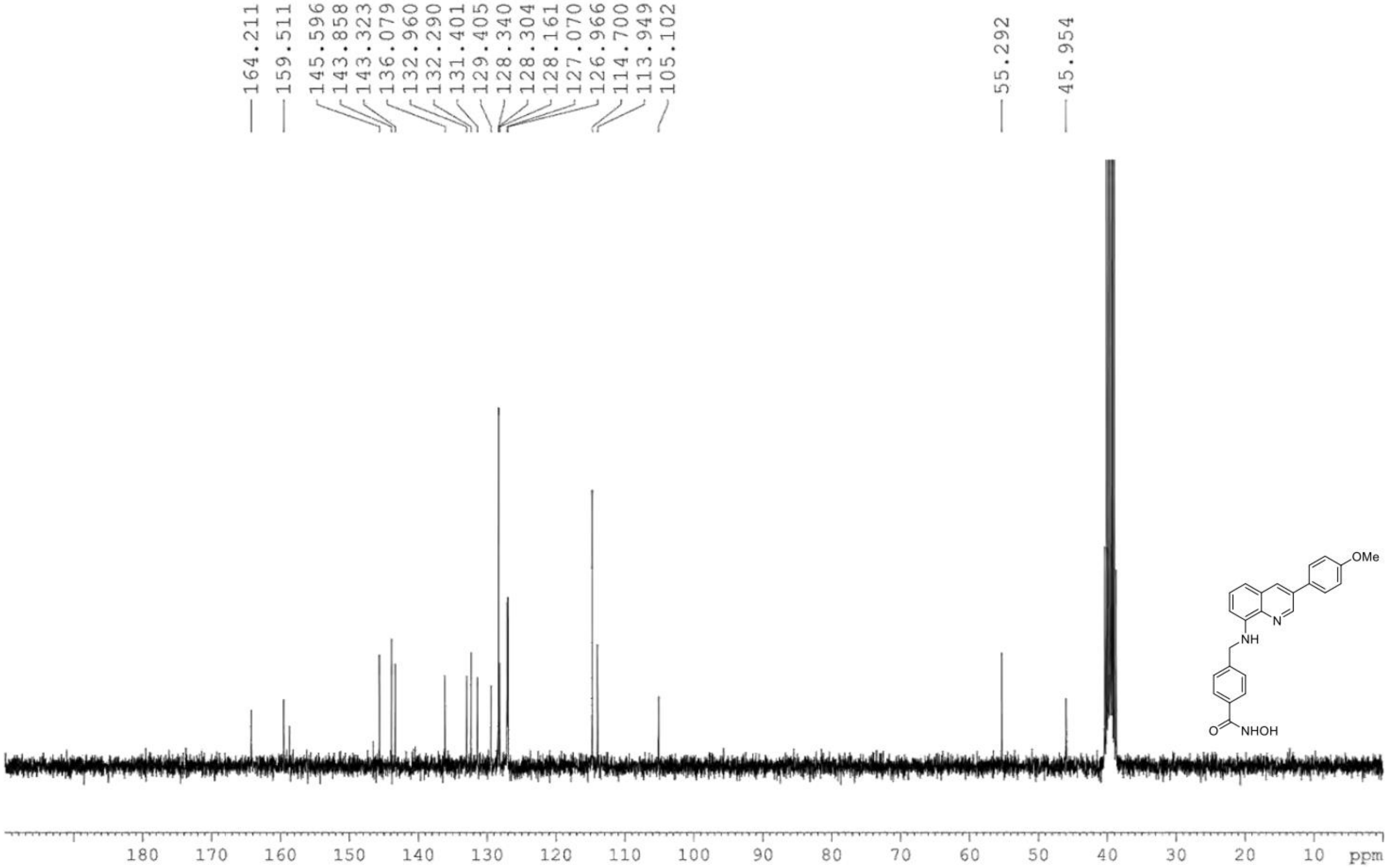

^{13}C Spectra for compound 21

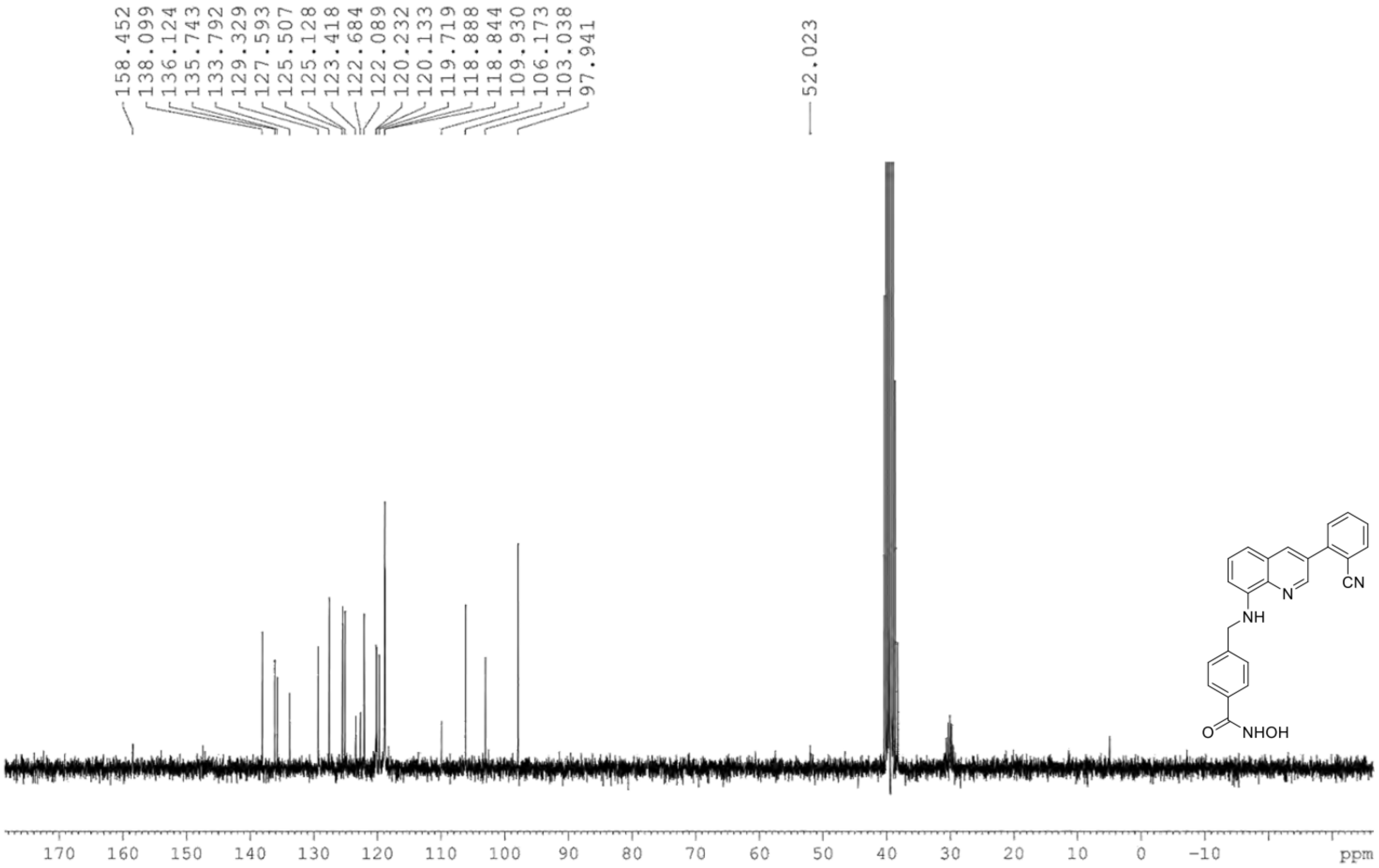

^{13}C Spectra for compound 22

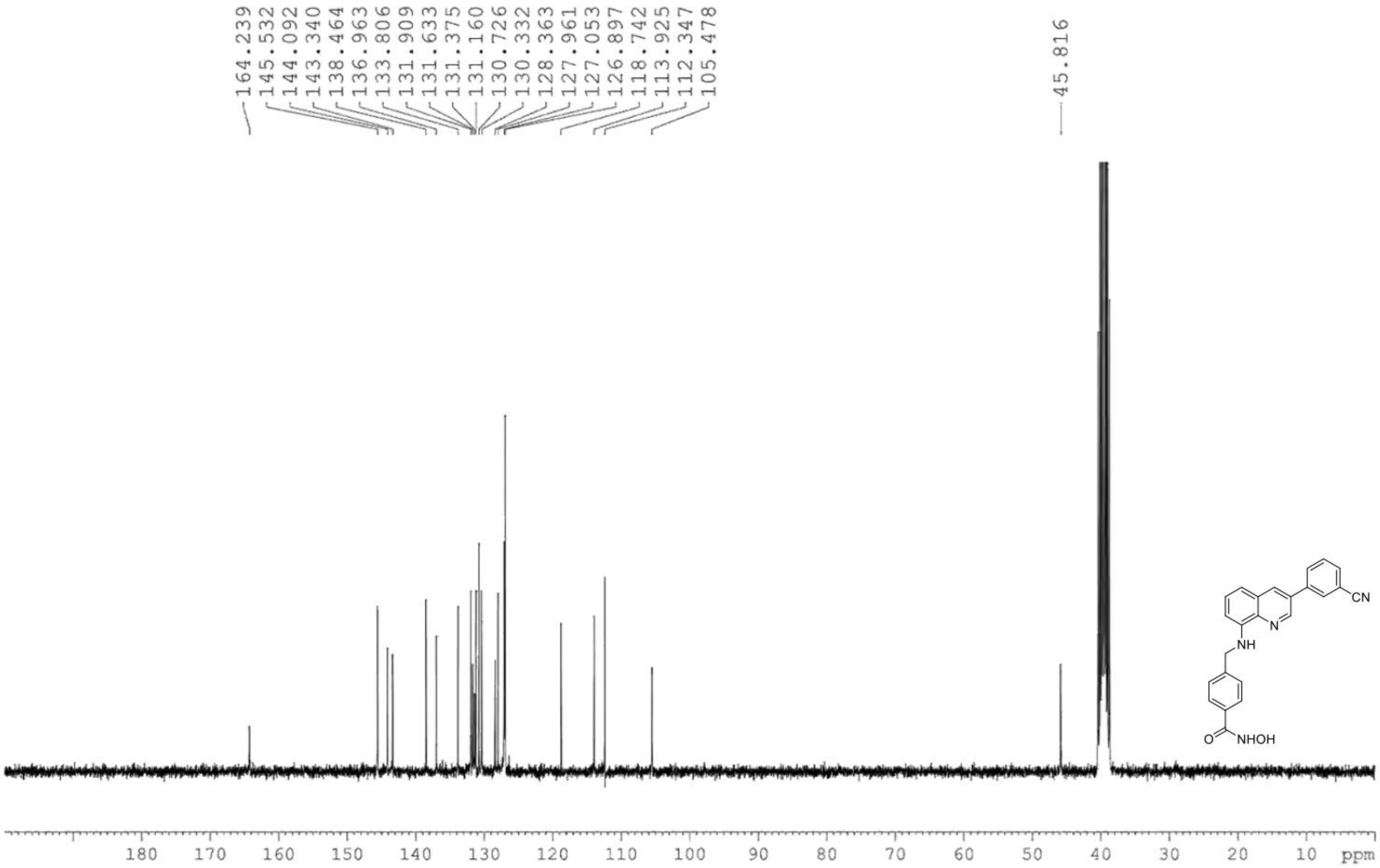

^{13}C Spectra for compound 23

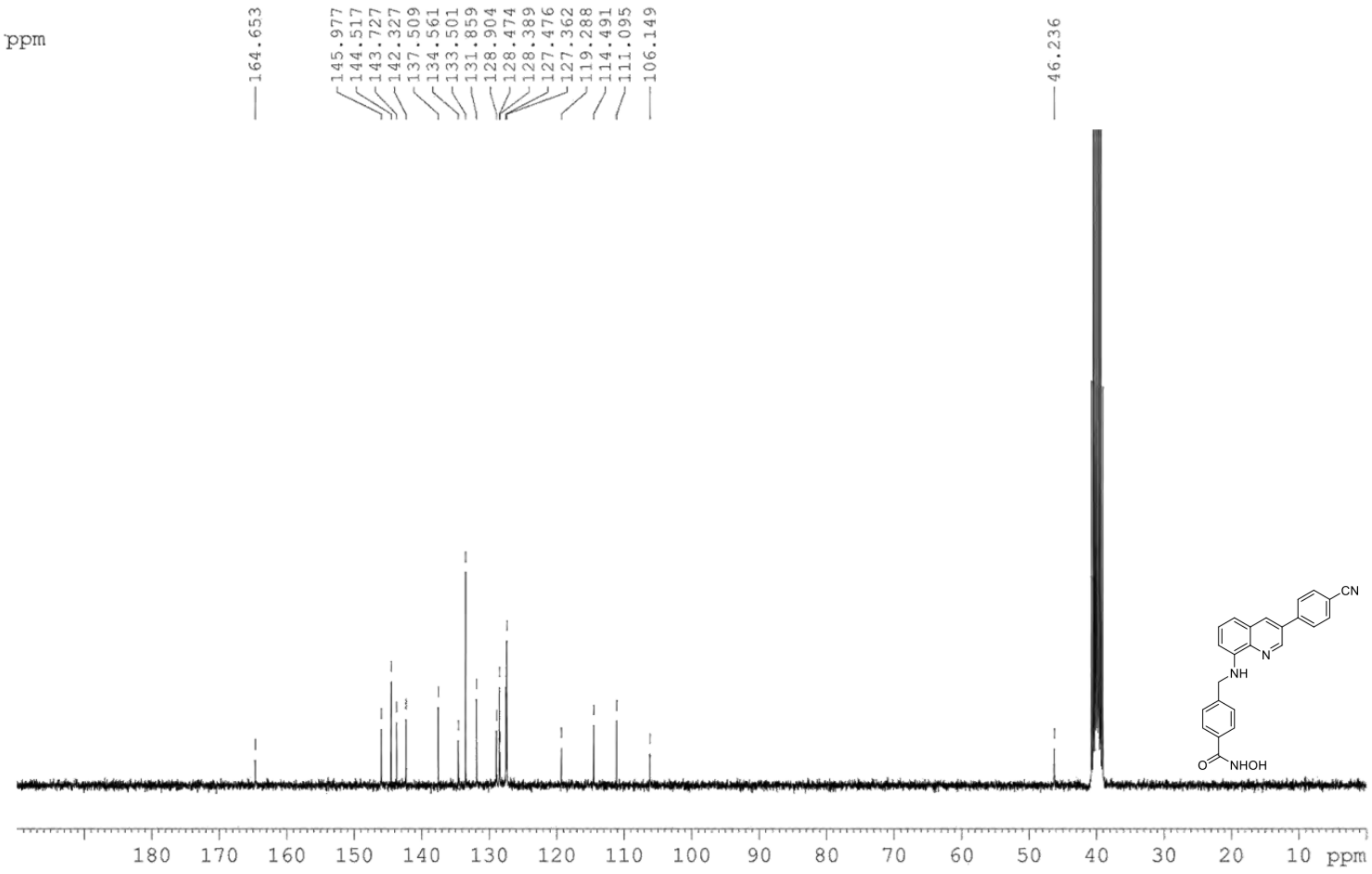

^{13}C Spectra for compound 24

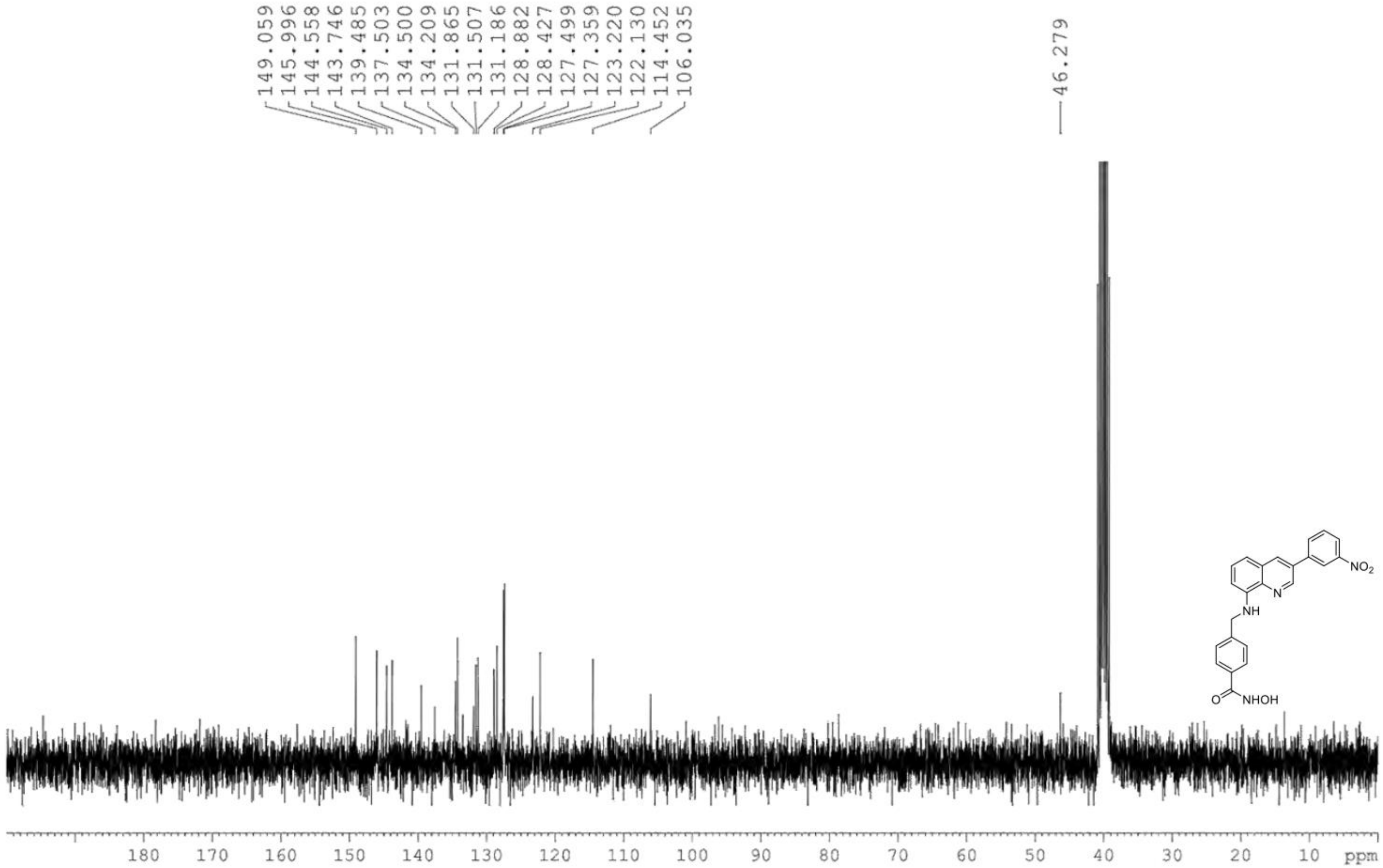

^{13}C Spectra for compound 25

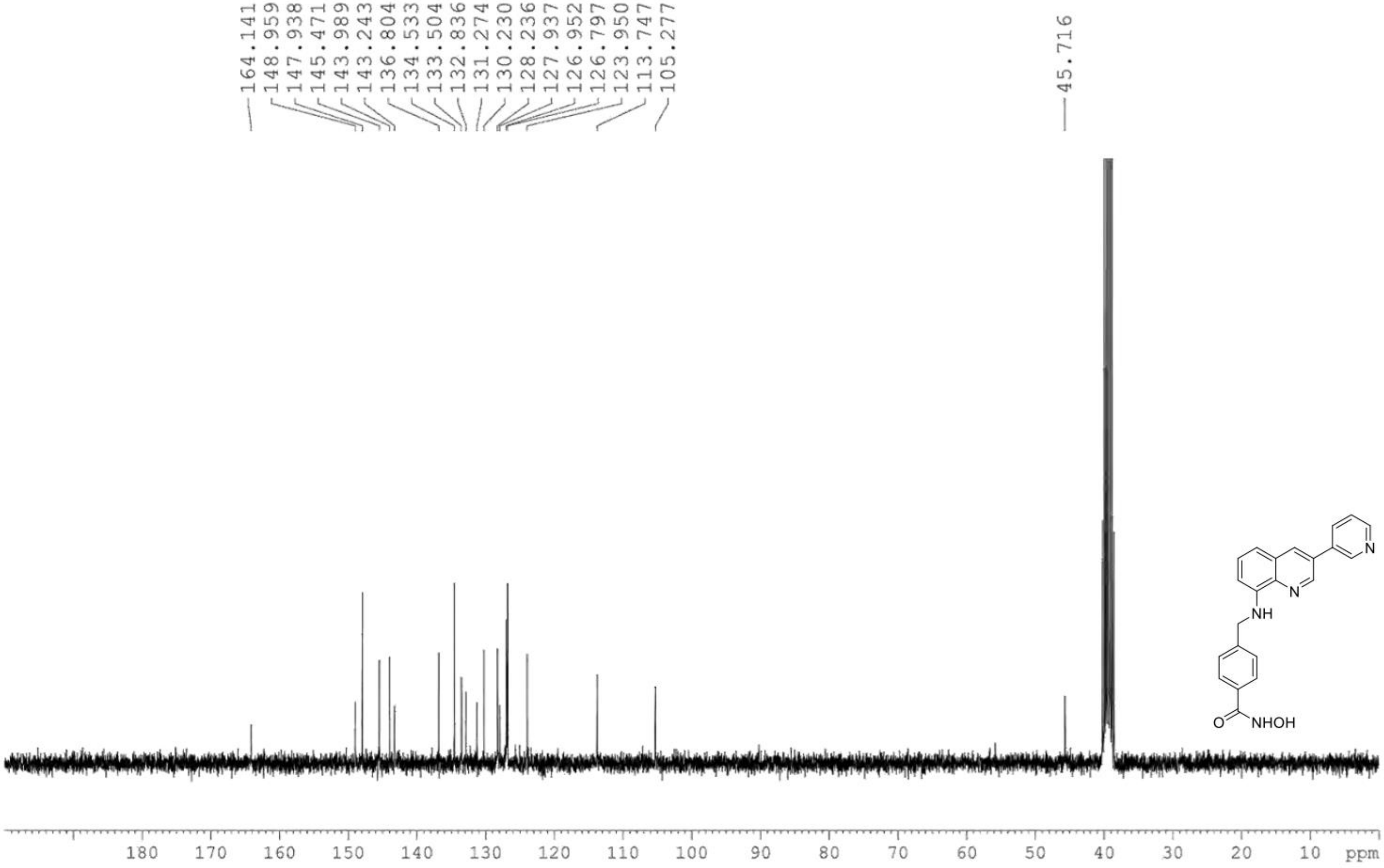

^{13}C Spectra for compound 26

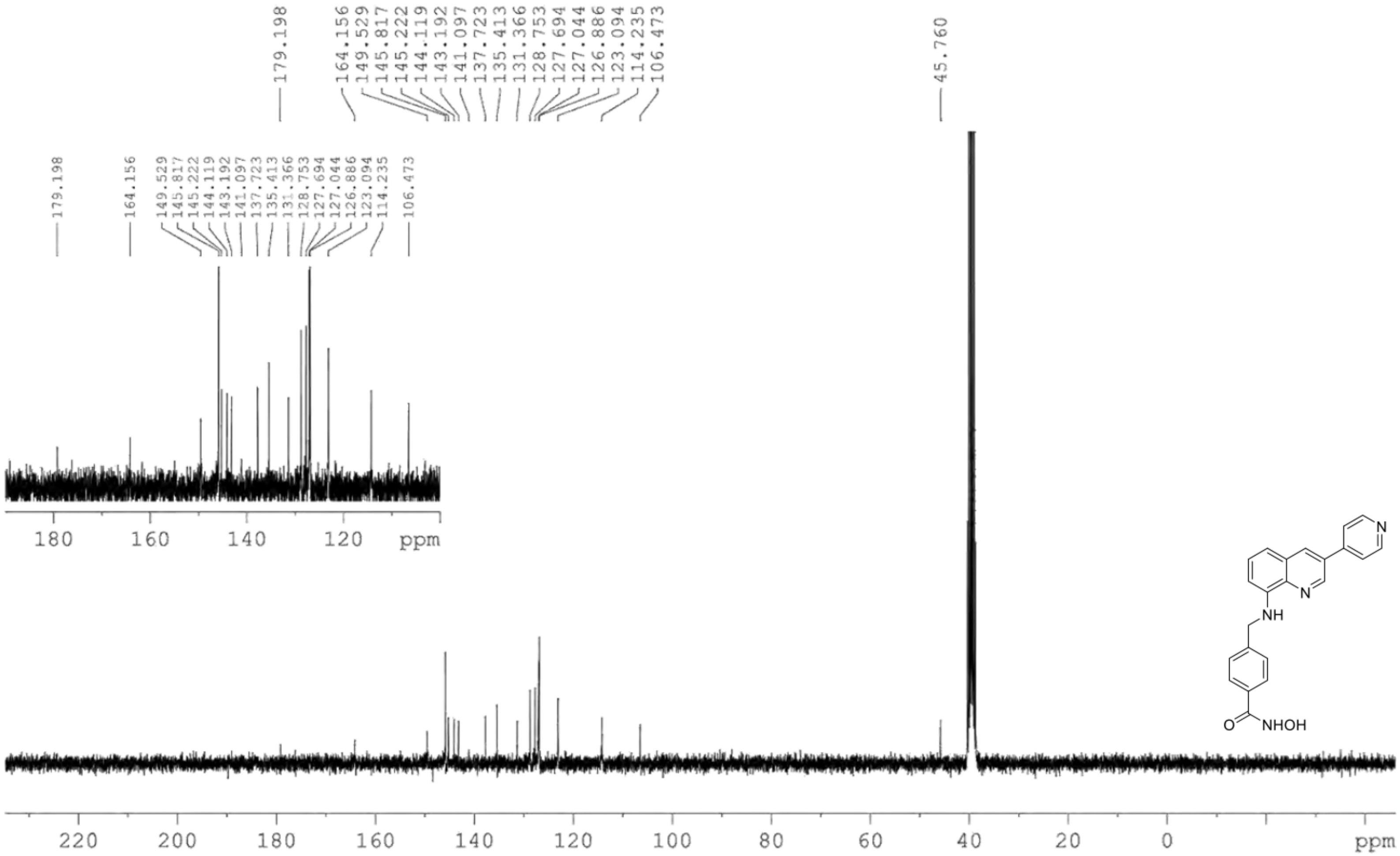

^{13}C Spectra for compound 27

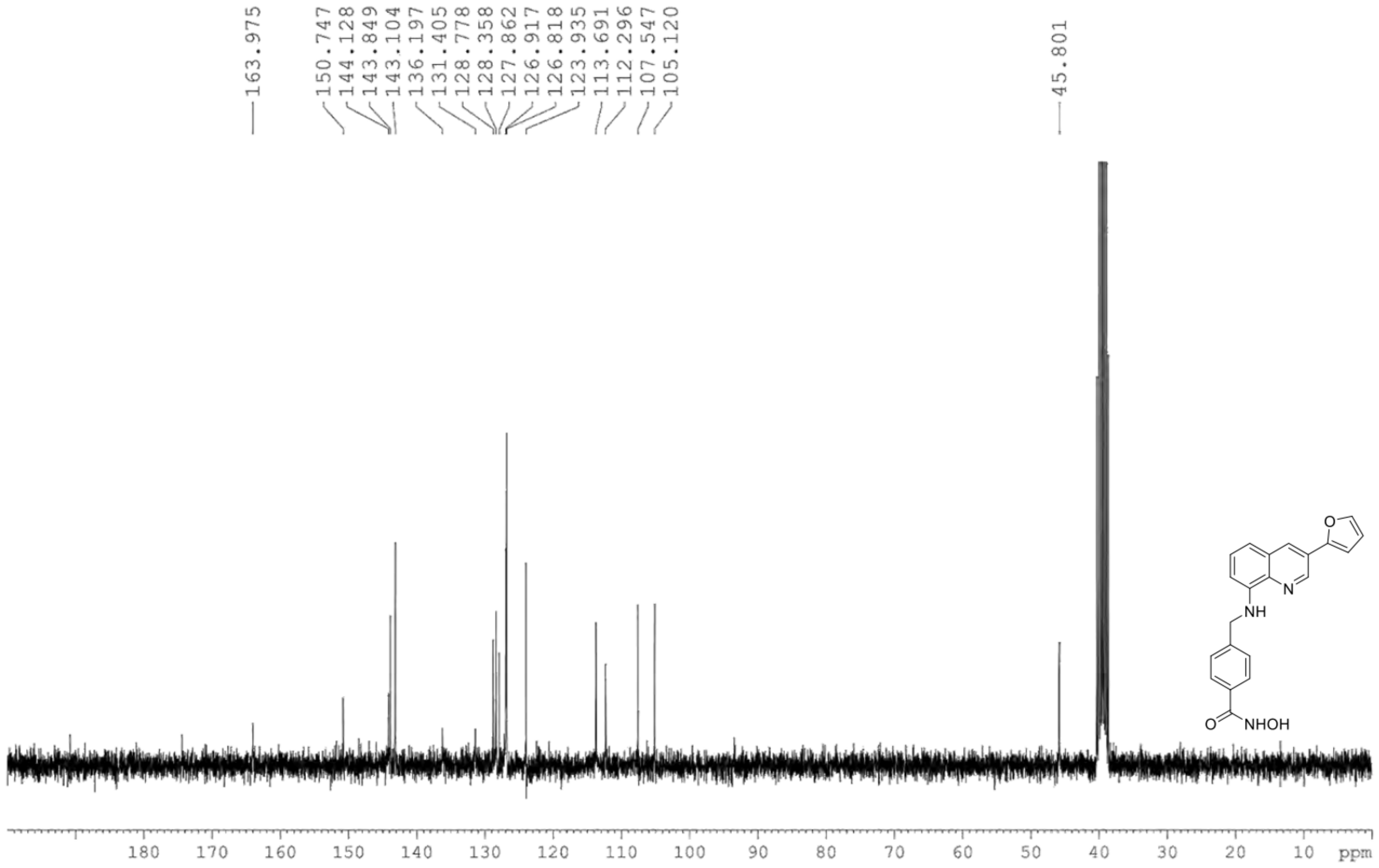

^{13}C Spectra for compound 28

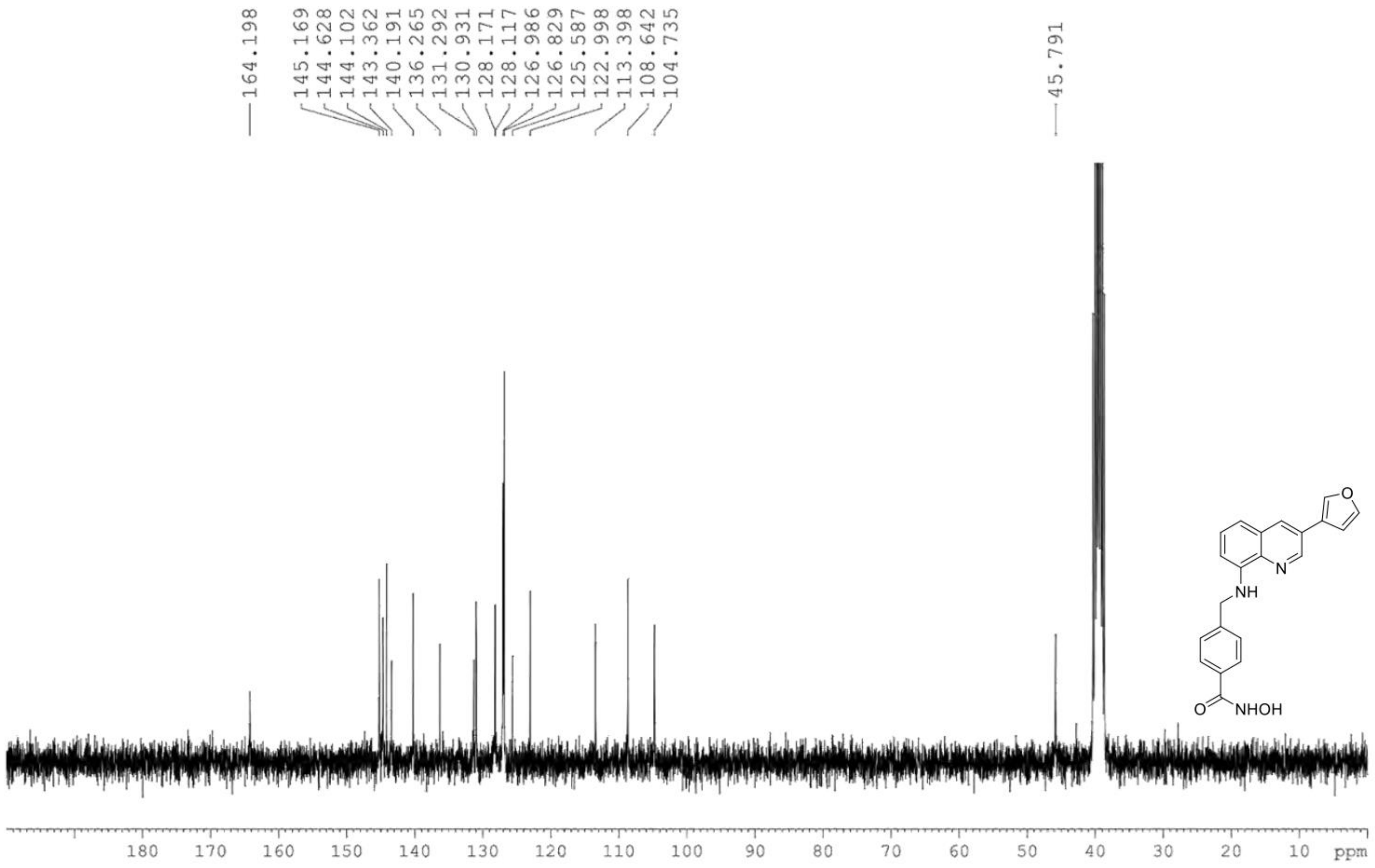

[13]C Spectra for compound 29

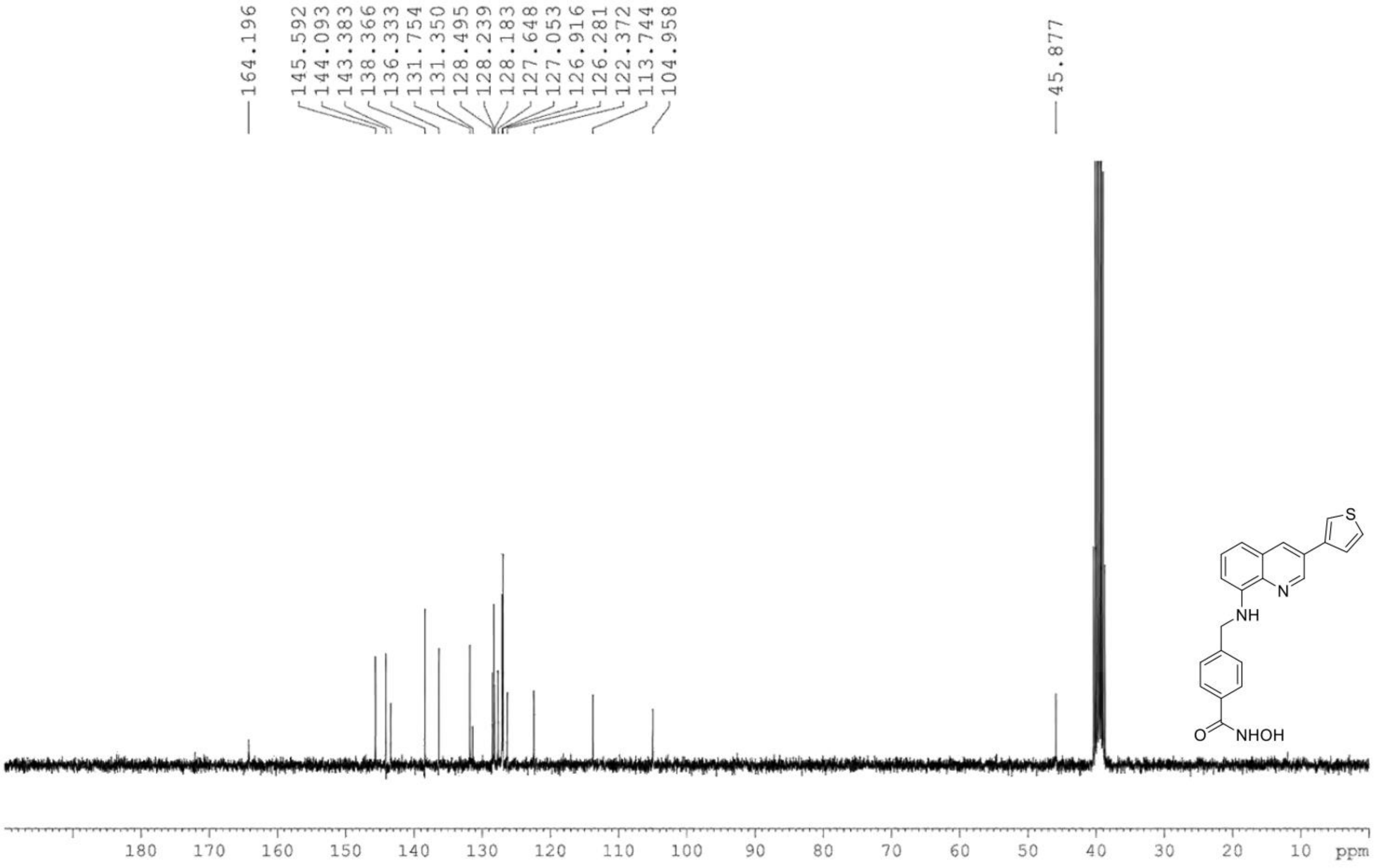

Supplement: Supplemental Material [file IENZ_A_1839446_SM2822.pdf]
